# Quantifying the Evolutionary Potential for Delta Smelt Persistence in a Warming Habitat

**DOI:** 10.1111/eva.70317

**Published:** 2026-09-01

**Authors:** Joanna S. Griffiths, Amanda J. Finger, Md. Moshiur Rahman, Brittany E. Davis, Tien‐Chieh Hung, Nann A. Fangue, Andrew Whitehead

**Affiliations:** ^1^ Department of Environmental Toxicology University of California Davis Davis California USA; ^2^ Conservation Biology Division, Northwest Fisheries Science Center, National Marine Fisheries Service National Oceanic and Atmospheric Administration Seattle Washington USA; ^3^ Department of Animal Science University of California Davis Davis California USA; ^4^ Department of Biological and Agricultural Engineering, Fish Conservation and Culture Laboratory University of California Davis Davis California USA; ^5^ Division of Integrated Science and Engineering, California Department of Water Resources West Sacramento California USA; ^6^ Department of Wildlife Fisheries and Conservation Biology University of California Davis Davis California USA

**Keywords:** conservation physiology, critical thermal maximum, domestication selection, ecophysiology, fish, genome‐wide association study, genomics, temperature

## Abstract

Long‐term persistence of managed species will depend, in part, on whether the species harbors the physiological or genetic potential to adjust to warming temperatures, and whether relevant genetic variation is modified by management practices. The critically endangered Delta Smelt ( *Hypomesus transpacificus* ) is intensively managed, but little is known about the presence of genetic variation for resistance to elevated temperature. Using a pedigree and whole genome sequencing data, we characterized the genetic basis of CTMax (as a metric of upper thermal tolerance) across control and elevated rearing temperatures, alongside covarying traits (body size and degree of hatchery ancestry). Warmer rearing temperatures increased CTMax through acclimation but also resulted in reduced additive genetic variation for the trait. We observed modest heritability for CTMax at rearing temperatures of 15°C and 18°C (0.26 and 0.16, respectively), but only a limited number of loci were identified that had consistent effects on CTMax across rearing temperatures. Instead, the genomic basis of thermal tolerance was highly dependent on rearing temperature (i.e., many loci detected with a GxE effect). This temperature‐dependent genomic architecture is consistent with our finding that additive genetic variation for CTMax was reduced under warmer rearing conditions, indicating a potential constraint on adaptive evolutionary change. The influence of domestication selection was indicated by changes in allele frequency, and divergence in upper thermal tolerance and plasticity, between low and high hatchery ancestry groups. Minimal overlap between loci associated with domestication and CTMax suggests that these traits possess separate genetic underpinnings. Knowledge of genetic variation supporting ecologically relevant physiological variation may be useful for captive management and may inform supplementation of fish to the wild in an ever‐warming environment.

## 1 Introduction

Climate change is exposing organisms to novel environments and causing detrimental physiological impacts. Resilience to sustained and extreme temperature events will be necessary for wild species to persist, where resilience may be achieved through physiological plasticity (acclimation ability) and evolutionary adaptation. Rapid adjustments may be achieved through plasticity (Bradshaw 1965), but plasticity may be insufficient for maintaining fitness in warming environments, such that evolution is required. A species' capacity to evolve depends on the presence and nature of heritable genetic variation underlying putatively adaptive traits (Lynch and Walsh 1998). Consequently, preserving genetic diversity, especially that fraction of diversity underlying adaptive traits, is a crucial objective for the conservation and management of wild species (Holderegger et al. 2006; Mable 2019).

Genetic and environmental influences can each contribute and interact to affect phenotypic variation, where the genetic contribution can be environmentally dependent. For example, environmental stress can increase available genetic variation (e.g., expose cryptic genetic variation) or decrease genetic variation (e.g., when species approach physiological limits) (Jenkins et al. 1997; Mcguigan et al. 2010; McGuigan and Sgrò 2009). Therefore, it is best to examine genetic variation across multiple ecologically relevant conditions. Evolutionary adaptation will also depend on available additive genetic variation for phenotypic plasticity, where genotypes may have variable effects on phenotype across environmental conditions: genotype‐by‐environment interactions (GxE) (Via and Lande 1985). Another consideration is how the pace of evolutionary change may be further shaped by genetic correlations between traits, which can either constrain or facilitate evolutionary adaptation and may further depend on environmental context (Etterson and Shaw 2001; Lande and Arnold 1983).

Quantifying the multi‐faceted drivers of evolutionary change is particularly important for at‐risk species supported by conservation hatcheries or breeding programs where the primary objective is to supplement wild populations (Bradford et al. 2025; Fraser 2008). Populations maintained in captive environments are more susceptible to losing genetic diversity due to strong genetic drift and small effective population sizes (Fiumera et al. 2004; Hedrick 2001). Consequently, genetic bottlenecks may constrain evolutionary potential once fish are released back into the wild (Laikre et al. 2010; Lorenzen et al. 2012). These risks may be prevented or managed by genetically informed breeding that reduces the influence of genetic drift. However, captive environments can also impose strong selective pressures (intentionally or unintentionally), favoring traits like disease resistance, high yields, and an ability to live in high densities that can further erode genetic diversity.

Preserving genetic diversity through genetically informed breeding and gene flow with wild individuals has been a top priority in the captive‐rearing program of the Delta Smelt ( *Hypomesus transpacificus* ), a critically endangered osmerid fish (California Natural Diversity Database (CNDDB) 2024; NatureServe 2014) for which experimental releases of hatchery fish back into the wild are already underway (Baerwald et al. 2023; Davis et al. 2024; Hudson et al. 2026; T. Hung et al. 2019; U.S. Fish and Wildlife Service 2020). The population decline and eventual crash in the Sacramento‐San Joaquin Delta (Delta) was the consequence of several factors, including rising temperatures and salinities, competition with non‐native species, exposure to environmental pollution, and water management conflicts (Yanagitsuru et al. 2022). Delta Smelt have lower thermal tolerance limits compared to other native and non‐native species in the Delta (Davis et al. 2019; Hung et al. 2022; Swanson et al. 2000), potentially further disadvantaging them relative to competitors (Halverson et al. 2022). Field experiments involving caged Delta Smelt have demonstrated that high mortality rates are driven by warm summer temperatures with a lethality threshold of 26°C (Davis et al. 2024). Given that current conditions in the wild already reach these critical limits, it is imperative to assess the extent of genetic variation for thermal resilience in captive Delta Smelt, which will determine the species' capacity to adapt to a warming climate. This assessment is especially critical across fish with different hatchery ancestries, as recent evidence suggests that domestication may have already induced heritable shifts in Delta Smelt thermal physiology (Griffiths et al. 2026).

Our study aimed to investigate the genetic variation and genomic architecture of thermal tolerance in captive Delta Smelt. We also explored the influence of rearing temperature, hatchery ancestry (domestication index, or DI), and body size (fork length) on the architecture of this trait. Finally, we tested whether patterns of genetic variation between low and high DI fish were consistent with the influence of domestication selection. To achieve these goals, we established families of Delta Smelt from a range of hatchery ancestries (low, medium, and high DI), reared them at both ambient and elevated temperatures, measured the upper thermal tolerance of ca. 3000 fish, and genotyped fish at thousands of loci genome‐wide. We tested for genotype–phenotype associations for multiple traits including CTMax and for trait associations that varied with rearing temperature (GxE) and compared these between fish with different degrees of hatchery ancestry.

## 2 Methods

### 2.1 Adult Spawning and Offspring Rearing

Adult Delta Smelt were obtained from the refuge population at the University of California Davis Fish Conservation and Culture Laboratory (FCCL). Since its inception in 2008, FCCL uses genetically informed single pair crosses to reduce inbreeding and incorporates wild broodstock (when available) to maintain genetic variation (Frankham 2010; Utter and Epifanio 2002). Hatchery ancestry within this refuge population is quantified using a domestication index (DI) which represents the number of generations an individual's genome has spent in captivity. Wild fish are assigned a DI of 0, and offspring produced from wild parents have a DI of 1 (Chase et al. 2024; Finger et al. 2018). Offspring DI is calculated as the parental mean DI plus 1. For the experiments reported here, adult Delta Smelt were spawned in 2021 using a modified North Carolina II breeding block design (Figure S1), with parental pairs selected to have genetic relatedness below 0.02 (mean relatedness of crossed parents was 0.003). Eggs and sperm were manually expressed and fertilization occurred in 290–500‐mL plastic bowls (freshwater, 16°C) (Lindberg et al. 2013). Across 13 experimental breeding blocks, eggs from two females were independently fertilized with sperm from five males, resulting in ten distinct fertilization events per block (Figure S1). A total of 15 males and 6 females were crossed within the same DI category; low (DI < 7), medium (DI 7–9), or high (DI > 10) (Figure S2). Due to low fertilization success, an additional 5 males and 2 females were crossed within the low DI category. A separate “mixed” DI group was also generated by crossing males and females spanning low, medium, and high DI categories.

At three days post‐fertilization (dpf), offspring from each cross were divided evenly between two rearing temperature treatments, with two replicate incubation columns at 15°C and two at 18°C (Figure S1), housed within two parallel recirculating aquaculture systems (Tsai et al. 2022). Offspring from different crosses were further pooled together based on DI category (low, medium, high, or mixed), resulting in 16 total incubation columns (four DI categories × two temperatures × two system replicates). For each family, between 10 and 60 embryos were stocked per treatment replicate. Families that did not produce enough embryos to meet the minimum per‐treatment allocation were excluded from the experiment. Hatching occurred at approximately 10 dpf, after which larvae were transferred to 38 L trays; at 40 dpf, larvae were moved into 133‐L tanks and reared until 143–151 dpf to complete all experiments. Fish were maintained in freshwater, under a natural photoperiod, and fed live prey consisting of cultured rotifers (*Brachionus plicatus*) and brine shrimp nauplii ( *Artemia franciscana* ) (Lindberg et al. 2013). All animal handling, care, and experimental procedures were approved (UC Davis IACUC Protocol #21915).

### 2.2 Thermal Tolerance Measurements

Upper thermal tolerance was assessed using a modified critical thermal maximum (CTMax) method (Beitinger et al. 2000) that was consistent and repeatable (Griffiths et al. 2026) in larval Delta Smelt aged 71–79 dpf. Preliminary trials described in Griffiths et al. (2026) demonstrated that larval CTMax measures have significant post‐recovery mortality in Delta Smelt (89%); however, mortality was independent of CTMax values and experimenter. CTMax was measured in 195 individuals from each replicate group (*n* ~ 390 per treatment [4 DI groups × 2 rearing temperatures]; 3120 fish total). Individuals were placed separately into 30‐mL opaque cups equipped with gentle air bubblers for mixing and aeration. Cups were then submerged in temperature‐controlled water baths, where water temperature was increased at a constant rate of 0.3°C min^−1^ until fish reached loss of equilibrium (LOE), a standard endpoint for determining thermal tolerance limits (Beitinger et al. 2000; Davis et al. 2019; Jeffries et al. 2016; Komoroske et al. 2014). The water temperature at LOE was recorded using a calibrated immersion thermometer.

A linear mixed‐effects ANOVA (R package lme4 v1.1–35.1 (Bates et al. 2014) and car v3.1–2 (Fox and Weisberg 2019)), was used to test for the fixed effects of DI (low, medium, or high), rearing temperature (15°C or 18°C), fork length, and their interaction on CTMax (the response variable), with trial number as a random effect, and tank/treatment group nested within the recirculating system as a random effect (Figure S1). We also ran a linear mixed‐effects ANOVA for fork length, with DI and rearing temperature as fixed effects and their interaction. To meet the assumptions of the ANOVA, we performed an orderNorm transformation on CTMax and fork length data (bestNormalize v1.9.1; Peterson 2021).

### 2.3 Family‐Level Survival

Using the pedigree from genotyped offspring (methodology described below), we were able to estimate the percent family survival based on the fraction of the starting number of fertilized embryos that survived to 71–79 dpf when we collected fish for CTMax measurements. We performed a linear mixed‐effect ANOVA (R packages lme4 v1.1–35.1 and car v3.1–2), with family survival (percent alive) as the response variable, and DI and rearing temperature as fixed effects. The random effects in the model were the family ID and tanks that were nested within the recirculating system. To investigate how survival varied between the two rearing temperatures, we performed an ANOVA with rearing temperature and family ID as fixed effects, and tanks nested within the recirculating system as a random effect. Finally, we investigated whether family survival correlated with CTMax by estimating the mean CTMax per family per replicate. We performed a linear mixed‐effect ANOVA with mean family CTMax as the response variable, mean family survival as a fixed effect, and tanks nested within the recirculating system as random effects. To meet the assumptions of the ANOVA, we performed a Box‐Cox transformation on survival data (bestNormalize v1.9.1; Peterson 2021).

### 2.4 DNA Extraction and Library Preparation

Fin clips (2–4 mm^2^) were sampled from 87 parents following spawning, and from 3085 of their offspring following CTMax trials. DNA extractions were performed using Agencourt Ampure XP beads as described in (Ali et al. 2016). DNA yield was assessed using a plate reader (BioTek Instruments, Winooski, VT). We normalized all samples to 2 ng/uL. We constructed 3172 whole‐genome sequencing libraries using a modified Twist 96‐Plex Library Preparation protocol (Twist Bioscience, San Francisco, CA). We targeted an insert size of 400 bp and library quality was assessed using a Bioanalyzer (Agilent, Santa Clara, CA). We created 5 pools of 560–650 offspring samples per pool. Each pool was sequenced on a single lane of NovaSeq S4 PE150 (target depth 1–2×). One pool of all 87 parent samples was sequenced across 4 lanes of NovaSeq S4 PE150 (target depth 30×).

### 2.5 Reference Panel Generation

To enable offspring genotype imputation, we generated a reference panel using the 87 high coverage parent samples. We used the snpArcher pipeline with snakemake v5.32.2 (https://snparcher.readthedocs.io/en/latest/index.html) to create a VCF file of the 87 parent samples. This pipeline performs quality control, read mapping, and variant calling. We mapped reads to the *Hypomesus transpacificus* reference genome (GCF_021917145.1) and called SNPs using GATK and applied the recommended GATK filtering (McKenna et al. 2010).

We applied additional filters to retain SNPs with a depth between 2 and 100× using vcftools v1.14 (Danecek et al. 2011) and biallelic SNPs using bcftools v1.14 (Danecek et al. 2021). We required SNPs to be genotyped in at least 50% of parents and to be in Hardy–Weinberg Equilibrium. Finally, we applied a minor allele frequency (MAF) cutoff of 2% using vcftools (v1.14) based on our imputation accuracy (see “*Imputation Validation”* section below). We removed sites with imputation quality < 0.065 (i.e., the info field generated by GLIMPSE). The filtered VCF file consisted of 1,300,115 SNPs. We used the program SHAPEIT5 v5.1.1 (Hofmeister et al. 2023) to phase all samples at common variants (MAF ≥ 0.1%). SHAPEIT5 was run with a recombination rate of 1 cM per Mb and an expected error rate in the phase informative reads of 0.0001 (the default setting).

### 2.6 Offspring Genotype Imputation and Pedigree Generation

We used the same snpArcher pipeline to generate aligned bam files for the 3085 offspring samples. We first estimated genotype likelihoods with bcftools v1.14 mpileup using a list of variant positions extracted from the parent reference panel. We then used the phased parent VCF as a reference panel to impute all low‐coverage sequenced offspring samples based on genotype likelihoods. Following recommended guidelines with the GLIMPSE v1.1.1 software (Rubinacci et al. 2021), we first split the phased reference panel into smaller chunks (2 Mb chunks with a buffer size of 200Kb). To impute genotypes, we ran GLIMPSE_phase with 15 iterations and then ligated chunks together using GLIMPSE_ligate. Finally, we generated a pedigree using genotyped parents and imputed offspring in the program AlphaAssign with a parentage assignment threshold corresponding to a 99.99% posterior probability (Whalen et al. 2018).

### 2.7 Imputation Validation

To validate imputation accuracy, we down‐sampled the mapped reads from one of our parent individuals from 30× to 1× coverage using samtools v1.14 (Danecek et al. 2021) and imputed this individual using the phased reference panel. We first re‐created our phased reference panel after removing this individual from the VCF file and followed the same methods as above for phasing. Next, we computed genotype likelihoods using bcftools mpileup v1.1.4 and imputed genotypes using GLIMPSE_phase for this down‐sampled parent as we did for the low‐coverage offspring samples. We computed the r2 correlation between imputed dosages (in MAF bins) and highly confident genotype calls. We used MAF bins of 0.00000, 0.00100, 0.00200, 0.00500, 0.01000, 0.05000, 0.10000, 0.20000, 0.50000. We were able to accurately impute variants up to ~2% MAF (*r* ^2^ = 0.8–0.85; Figure S3), resulting in a MAF cut‐off of 2% in the phased reference panel VCF for a total of 1,300,115 SNPs.

### 2.8 Quantitative Genetic Estimates

To quantify standing additive genetic variation and the capacity to evolve to warming temperatures, we estimated variance components for CTMax and fork length size using an animal model; a mixed model that uses a pedigree to estimate the relative contributions of genetic and environmental components on phenotypic variation (Kruuk 2004). Specifically, we used a generalized linear mixed model (GLMM) using Markov chain Monte Carlo (MCMC) in the R package MCMCglmm (Hadfield 2010) in R v.4.5.0. Using the pedigree generated from the genotyped parents and imputed offspring, we tested different model parameters to estimate variance components using DIC score comparisons for random effects (rearing temperature, dam ID, and fork length). In animal models, potential confounders (e.g., experimental system) are accounted for by including them as fixed effects and were included in the model if the 95% Highest Posterior Density (HPD) intervals for the posterior distribution for the fixed variables did not overlap zero. Our final model for estimating variance components for CTMax and fork length included experimental system as a fixed effect and rearing temperature as a random effect. Rearing temperature was set as a covariate random effect in the model so we could further partition the additive genetic variation by rearing temperature as a random interaction so that each animal has a separate effect for each rearing temperature level. This allowed us to directly compare variance components and heritability across different rearing temperatures in a single model. We ran these models with all fish, as well as separate identical models with the data subset by each DI category (low, medium, and high). Given that maternal effects have been shown to influence size of offspring in other species (Moore et al. 2019; Paul et al. 2023), we ran an additional model for fork length with dam ID as an additional random effect even though it was not statistically supported in model selection criteria.

To assess the extent to which additive genetic effects underlying thermal tolerance were conserved across rearing temperatures, we calculated additive co‐variation and genetic correlation for family‐level mean CTMax measured at 15°C and 18°C. We fit a multivariate model using full‐sibling family mean CTMax measured at 15°C and 18°C as co‐varying traits. In this model, tank replicate was included as a fixed effect. This approach enabled us to directly estimate the genetic correlation between family genotype and CTMax performance at 15°C vs. 18°C rearing. A correlation near 0 indicates that thermal performance at 15°C does not predict performance at 18°C, which suggests that there is some re‐ranking of genotype performance at each rearing temperature.

To quantify genetic correlations among traits and estimate additive genetic co‐variation, we fit two additional multivariate animal models. Phenotypic correlations arise from multiple sources, including shared environments, maternal effects, and additive genetic covariance, whereas genetic correlations specifically reflect shared additive genetic influences between traits, arising from pleiotropy or physical linkage among loci. Therefore, we fit a multivariate model with CTMax and fork length as the co‐varying response traits, and a second model with CTMax and numerical DI as the co‐varying response traits. In both models, experimental system was included as a fixed effect and rearing temperature as a random effect. These analyses allowed us to assess whether genetic variation underlying thermal tolerance covaries with body size or DI across the two rearing temperatures.

All models were run for 500,000 iterations, a burn‐in of 1000, and a thinning interval of 500, resulting in a posterior sample size of 998 estimates. Autocorrelation values for the parameters were near zero, confirming that convergence occurred and there were no trends in the parameters over successive generations of the model. Heritability was calculated as the ratio of observed additive genetic variance to total phenotypic variance. We also calculated evolvability for CTMax and fork length as the ratio of observed additive genetic variance to the squared mean of the trait value. Genetic correlation between two traits was calculated as the ratio of observed additive genetic co‐variation to the square root of additive genetic variation for the two traits multiplied together. All quantitative genetic estimates are reported as posterior modes with the 95% HPD intervals.

### 2.9 Genomic Architecture of Thermal Tolerance and Fork Length

We performed a genome‐wide association study (GWAS) for thermal tolerance and fork length using the program GEMMA v0.98.3 (Zhou and Stephens 2012). A relatedness matrix was computed in GEMMA with the centered method. To test for the main effect of genotypes on CTMax and fork length, we ran a linear mixed model with the trait measurement as the response variable and rearing temperature and experimental system as covariates. We were unable to include fork length as an additional covariate in the model given that it is highly correlated with the temperature treatments in the experimental system covariate parameter (i.e., growth was faster, and therefore size was larger, in fish reared at elevated temperature). GEMMA also reports the proportion of variance explained by genotypes (“SNP heritability”) for the trait from the linear mixed model. To detect genotypic effects on CTMax that depended on the rearing temperature (GxE), we ran a second linear mixed model with the “‐gxe” flag. The linear model controls for both the SNP main effect and environmental (rearing temperature) main effect, while testing for the interaction (i.e., genetic effect on CTMax or fork length that is temperature‐dependent). We plotted the results of the linear models as a manhattan plot (R package qqman) and applied a genome‐wide and chromosome‐level Bonferroni correction.

### 2.10 Signatures of Domestication Selection

We investigated evidence for genomic divergence between low and high domesticated fish using F_ST_ and GWAS approaches. We measured pairwise F_ST_ (Weir methodology, window size of 10,000 bp, sliding window of 5000 bp) between low and high DI progenitor fish (*N* = 31 and 29, respectively) using vcftools v0.1.16 (Danecek et al. 2011). We ran a permutation test to determine whether F_ST_ was higher than expected by chance by randomly assigning individuals into two groups (1000 random permutations) as in Griffiths et al. (2026).

We also explored genomic associations with hatchery ancestry in the offspring dataset using a GWAS on numerical DI with methods similar to those described above for GWAS of thermal tolerance and fork length. We ran a linear mixed model in GEMMA with the numerical DI as the response variable and experimental system as a covariate. We plotted the results of the linear models as a manhattan plot (R package qqman) and applied a genome‐wide and chromosome‐level Bonferroni correction.

### 2.11 Functional Annotation and Enrichment of Candidate SNPs

We used the program LD‐annot v0.4 (Prunier et al. 2019) with *r* ^2^ = 0.8 to identify gene regions and their annotations that are in linkage disequilibrium (LD) with SNPs statistically associated with CTMax, fork length, and DI GWAS analyses, and the F_ST_ analysis. Gene Ontology (GO) terms for each gene were identified by matching Delta Smelt gene names with those from the zebrafish annotation database in ensembl using the R package biomaRt v2.46.3 (Durinck et al. 2005, 2009). GO enrichment analysis was performed using the R package topGO v2.42.0 (Alexa and Rahnenführer 2007), with the background gene set defined as genes in LD with SNPs from the filtered progenitor VCF (23,835 genes). We used a Fisher's Exact test (*p* < 0.05) and retained GO terms that were represented by more than one significant gene.

To further explore the potential functions and downstream effects of genes associated with significant SNPs in the GWAS and F_ST_, we investigated whether these genes overlapped with those that were altered in their transcription or methylation by rearing temperature or DI; in a companion study, we had characterized differentially expressed genes (DEGs) and differentially methylated regions (DMRs) between 15°C and 18°C rearing temperatures and between low and high DI fish (Griffiths et al. 2026). For candidate genes identified in our GWAS for CTMax, we compared overlap with DEGs and DMRs due to rearing temperature. For candidate genes identified in our offspring GWAS for DI and our progenitor F_ST_, we compared overlap with DEGs and DMRs due to DI. We used a hypergeometric test (phyper function in base R v4.3.2) to determine whether the significant overlap was more than expected by chance.

### 2.12 Shared Genomic Basis

We evaluated whether traits shared a common genomic basis. We first evaluated overlap among sets of genes significantly associated with phenotypes in GWAS analyses, including the genetic‐only associations and temperature‐dependent associations for both CTMax and fork length, and the genetic‐only association with DI. To standardize among different statistical thresholds used in each GWAS, we selected the top 300 SNPs that were associated with each trait and identified genes in LD with those SNPs using LD‐annot v0.4 as described above. We also compared the top 300 SNPs from each GWAS to the low‐high progenitor F_ST_. We used two F_ST_ thresholds for comparisons: the top F_ST_ windows containing 300 SNPs and SNPs within windows with an F_ST_ above 0.1. We then used a hypergeometric test (implemented with the *phyper* function in base R v4.3.2) to assess whether the observed gene overlap exceeded expectations under random association, given the total number of genes (23,835) in LD to SNPs in the VCF. In addition, we applied a more stringent approach at the SNP level using the program PLACO+ (Park and Ray 2026). This approach also accounts for correlation among traits that arises from measurements on the same individuals. PLACO+ uses GWAS summary statistics and tests each SNP for joint association with both traits, returning a *p*‐value that indicates whether a SNP is significantly associated with both traits. We applied genome‐wide and chromosome‐level Bonferroni correction.

## 3 Results

### 3.1 Family Survival, CTMax, and Fork Length

A total of 130 families were generated, but 7 families had 0% fertilization success, and 14 families had poor fertilization success (defined by fewer than 40 total fertilized embryos). Out of the 3085 individuals genotyped, there were 104 families represented. Survival rates among families were similar across rearing temperatures (*F*_1,10.3_ = 0.92 *p* = 0.36; Figure S4A) and across DI categories (*F*_1,57_ = 0.67 *p* = 0.68; Figure S4A). We also investigated the plasticity of family survival under different rearing temperatures. However, percent survival rates were significantly variable among different families (*F*_1,102_ = 5.8 *p* < 2e‐16; Figure S4B) and there was significant variation in resilience to temperature among families (family‐level survival by rearing temperature interaction; *F*_1,94_ = 1.6 *p* = 0.005; Figure S4B).

**FIGURE 1:**
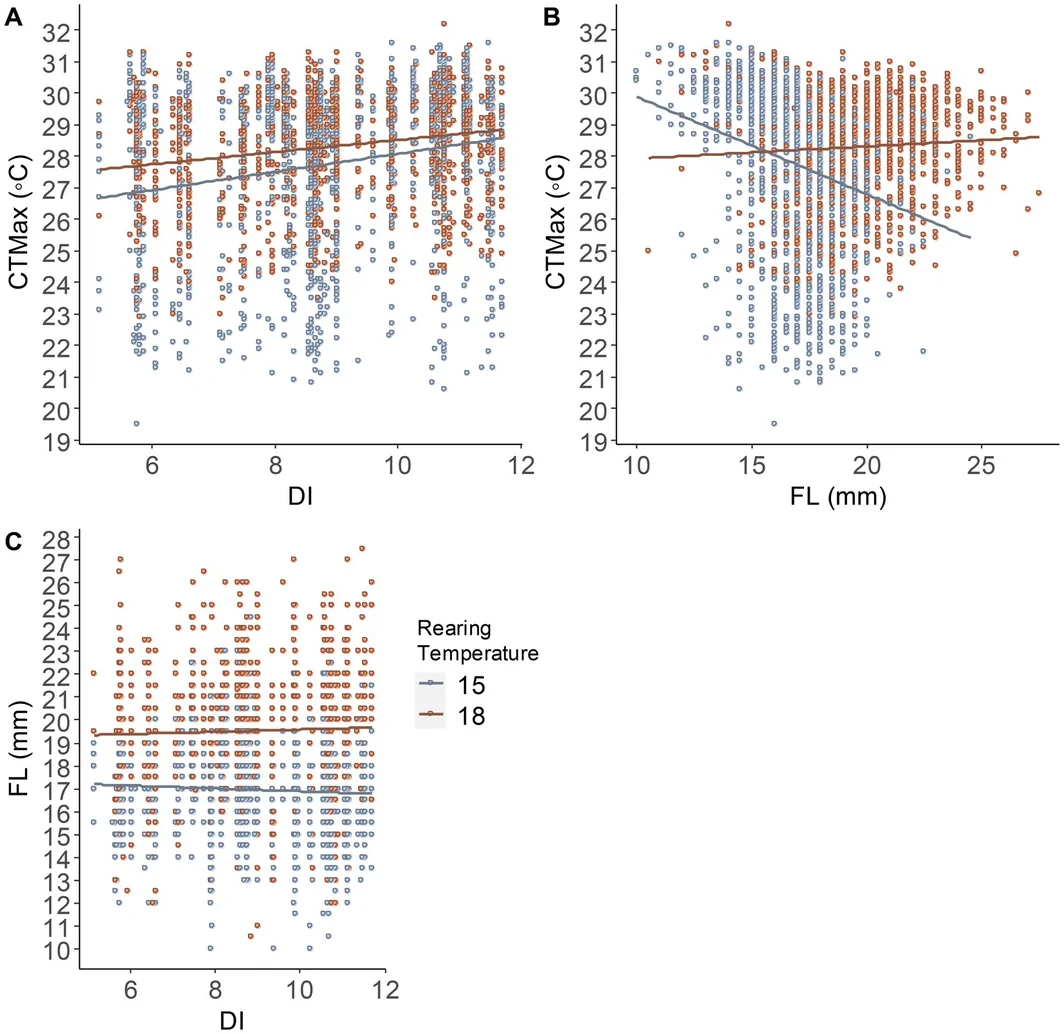
Critical Thermal Maximum (CTMax) for fish reared at either 15°C (blue) or 18°C (orange). (A) Fish reared at 18°C had a higher CTMax than fish reared at 15°C and high domestication index (DI) fish had higher CTMax than low DI fish. (B) Negative correlation was detected between CTMax and size (fork length, FL), where larger fish had lower CTMax. However, this correlation only existed for fish reared at 15°C. (C) Fish reared at 18°C were larger than fish reared at 15°C, but there was no correlation between fork length and DI.

Rearing fish at 18°C compared to 15°C significantly increased mean CTMax values by 0.6°C (*F*_1,17.4_ = 26.2 *p* = 7.9e‐5; Figure 1A) and higher DI fish had higher CTMax values (*F*_1,42.2_ = 23.9 *p* = 1.5e‐5; Figure 1A), with a 0.24°C increase in CTMax with each 1‐unit increase of DI (~1 generation spent in the hatchery). However, there was no interaction effect of rearing temperature and DI (*F*_1,55.4_ = 0.13 *p* = 0.71; Figure 1A). Fork length was also significantly correlated with CTMax values (*F*_1,2109_ = 119.6 *p* = 2.2e‐16; Figure 1B), with a significant interaction of rearing temperature and fork length (*F*_1,1682_ = 131.6 *p* = 2.2e‐16; Figure 1B); larger fish had a smaller CTMax value, but this effect was only present when fish were reared at 15°C. When investigating family‐level trait means, families with higher percent survival were also more likely to have higher mean CTMax (*F*_1,384_ = 22.4 *p* = 3.1e‐06; Figure S5).

Rearing fish at 18°C compared to 15°C significantly increased mean fork length by 2.6 mm (*F*_1,14.1_ = 17.5 *p* = 9.0e‐4; Figure 1C). DI had a statistically significant effect on fork length (*F*_1,1257_ = 49.2 *p* = 3.66e‐12; Figure 1C), but the slope is close to zero (0.15 mm of increase in FL per 1 unit change in DI), so we infer a very minor biological effect. Finally, there was no interaction effect of rearing temperature and DI on fork length (*F*_1,1119_ = 2.58 *p* = 0.23; Figure 1C).

### 3.2 Quantitative Genetics Estimates

We calculated the amount of additive genetic variation, heritability (h^2^), and evolvability for CTMax and fork length in Delta Smelt progeny. Additive genetic variation (V_A_) is the component of phenotypic variation that is attributable to the additive linear effects of individual alleles and represents the genetic component that responds to natural selection, while heritability expresses V_A_ as a proportion of phenotypic variation and is used to explain how much of the observed differences in a trait is genetically determined. Evolvability represents the capacity of a trait to evolve relative to the population's mean. Unlike heritability, which is influenced by environmental variance, evolvability is the mean‐scaled measurement of V_A_, providing a more direct representation of evolutionary potential (Hansen et al. 2011).

**FIGURE 2:**
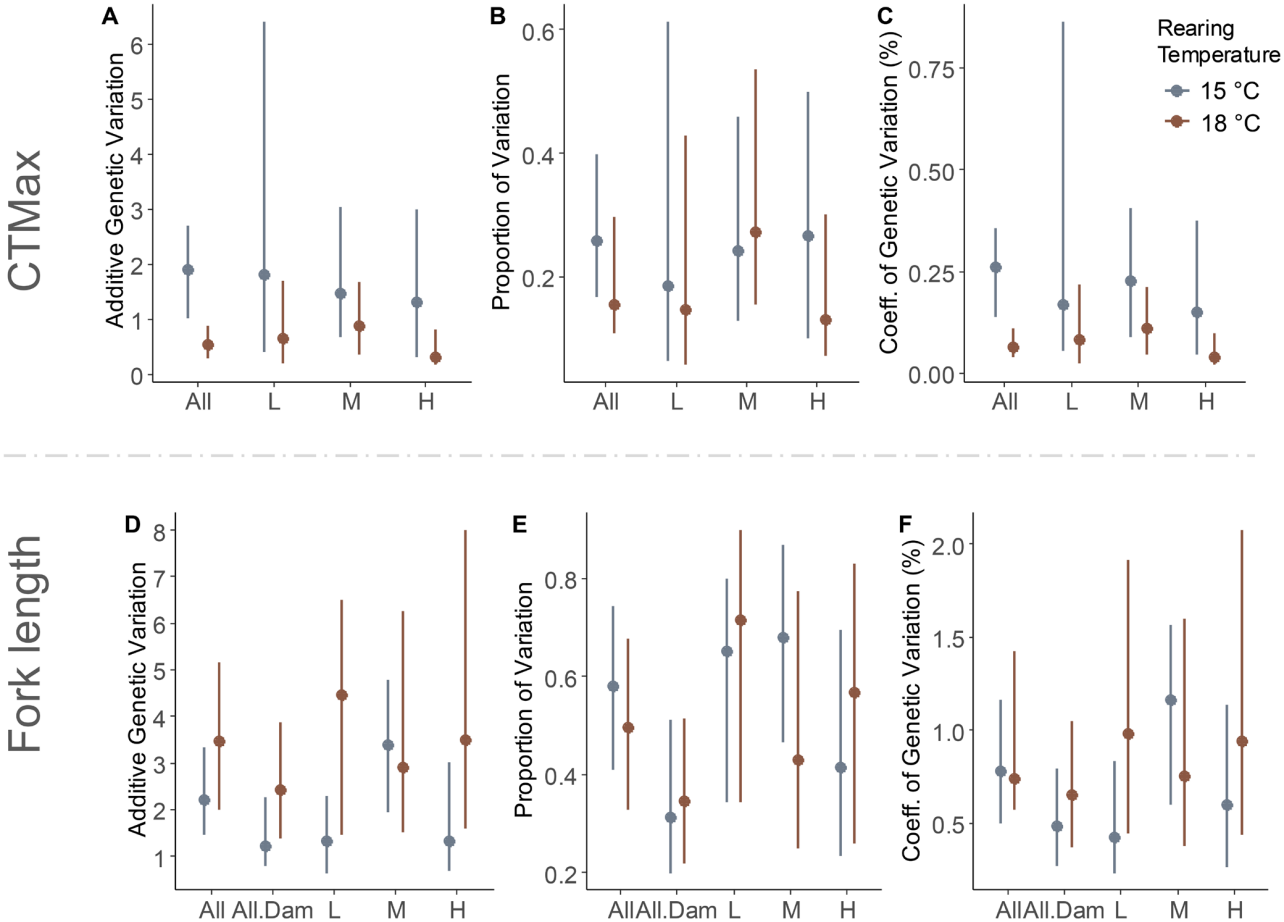
Additive genetic variation for CTMax (A) and fork length (D), proportion of phenotypic variation attributed to additive genetic variance (narrow‐sense heritability; *h* ^2^) for CTMax (B) and fork length (E), and evolvability (coefficient of genetic variation, %) for CTMax (C) and fork length (F). Points represent the mode of posterior distributions from the animal model with 95% confidence intervals displayed. Results are presented for the two models run with all fish (All: Without dam effects; All. Dam: With dam effects), and data subset by each domestication index (DI) category (L: Low, M: Medium, and H: High). A model with all fish and Dam ID set as a random effect for fork length are shown in (D, E, and F). Variation was further partitioned by fish reared at 15°C (blue) or 18°C (orange).

The additive genetic variation for thermal tolerance (CTMax) for fish reared at 15°C was significantly higher (1.91, 95% HPD: 1.02–2.70; Figure 2A) than for fish reared at 18°C (0.53, 95% HPD: 0.29–0.87; Figure 2A). Similarly, the estimates of evolvability for fish reared at 15°C were significantly higher (0.25%, 95% HPD: 0.14%–0.36%; Figure 2C) than for fish reared at 18°C (0.065%, 95% HPD: 0.04%–0.1%; Figure 2C). However, our estimates of heritability were not significantly different between fish reared at 15°C (0.26, 95% HPD: 0.17–0.40) and 18°C (0.16, 95% HPD: 0.11–0.30; Figure 2B). There was no significant difference in V_A_ for fork length between fish reared at 15°C (2.22, 95% HPD: 1.47–3.34; Figure 2D) and 18°C (3.48, 95% HPD: 1.99–5.16; Figure 2D). Similarly, there was no significant difference in heritability estimates for fork length between fish reared at 15°C (0.78, 95% HPD: 0.50–1.16; Figure 2E) and 18°C (0.74, 95% HPD: 0.57–1.42; Figure 2E), nor were there significant differences in evolvability for fork length in fish reared at 15°C (0.25%, 95% HPD: 0.14%–0.36%; Figure 2F) and at 18°C (0.065%, 95% HPD: 0.04%–0.1%; Figure 2F). When dam ID was included as a random effect in the model, posterior modes were lower but not significantly different than the model without dam effects (Figure 2B). For both CTMax and fork length traits, when the data were subset for each DI category, there were no significant differences in additive genetic variation, heritabilities, nor evolvability between fish reared at 15°C or 18°C, nor among different DI categories (Figure 2A,B). However, we note that the HPD intervals were large, suggesting that statistical power for detecting DI effects was low.

To assess the extent to which additive genetic effects underlying thermal tolerance were conserved across rearing temperatures, we calculated additive co‐variation and genetic correlation for family‐level mean CTMax measured at 15°C and 18°C. The additive co‐variation between family‐level CTMax at the two temperatures was significantly positive (0.30; 95% HPD 0.05–0.50) and the estimated genetic correlation was also positive (0.46; 95% HPD 0.08–0.73). However, we note that these two traits are not perfectly correlated indicating that the genetic ranking of thermal tolerance also changes in each rearing temperature (i.e., genotype‐by‐environment interaction).

We also calculated the genetic correlation between thermal tolerance and fork length and numerical DI (the shared genetic basis between two separate traits). We found a strong negative genetic correlation between CTMax and fork length when fish were reared at 15°C (−0.50, 95% HPD: −0.69 to −0.24), but no genetic correlation when fish were reared at 18°C (−0.04, 95% HPD: −0.32 to 0.28). However, we note that the HPD overlap slightly, suggesting that there is not a statistically significant difference in genetic correlations at each rearing temperature. We found a slightly positive genetic correlation between CTMax and numerical DI when fish were reared at 15°C (0.07, 95% HPD: 0.02–0.16) and 18°C (0.1, 95% HPD: 0.02–0.17). However, there was no difference in genetic correlations for CTMax and DI between the 15°C and 18°C rearing temperatures.

### 3.3 Genomic Architecture of Thermal Tolerance

**FIGURE 3:**
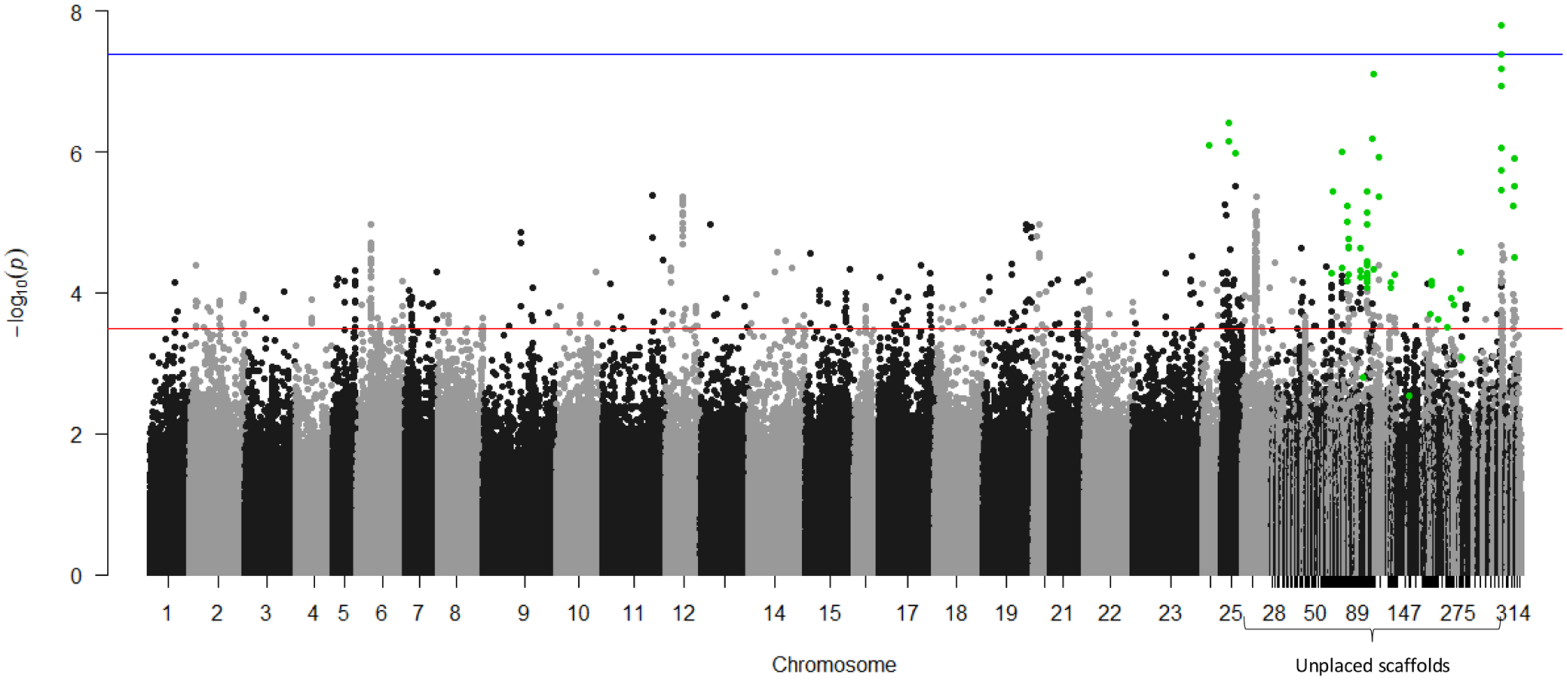
Genome‐wide association test (GWAS) for the effect of genotypic variation on CTMax scores for 3085 individual fish. Manhattan plot of *GEMMA* model results (−log *p*value) for SNPs associated with CTMax. The blue horizontal line represents the genome‐wide *p* = 0.05 significance threshold after Bonferroni correction. The red horizontal line at −log_10_ (*p*) ≥ 3.5 was chosen to encapsulate peaks of association for functional enrichment exploration. SNPs highlighted green are ones that pass the chromosome (or scaffold) *p* < 0.05 Bonferroni corrected significance threshold. Genes linked to SNPs highlighted in green and those above the red horizontal line were tested for GO enrichment.

Using a GWAS to identify loci underlying variation in thermal tolerance, we tested for the main effect of genotype on CTMax (while controlling for rearing temperature) and for temperature‐dependent genotype effects (GxE). The SNP heritability for the main effect of genotype was estimated at 0.14 (SE ±0.03), with a single genome‐wide significant SNP on an unplaced scaffold (Figure 3) that was in LD with two protein coding genes, the uncharacterized protein unc13bb and the chemokine protein ccl19b. Thermal tolerance is broadly recognized as a polygenic trait (Fuller et al. 2020; Gonen et al. 2024; Griffiths et al. 2020; Michalak et al. 2019; Tobler et al. 2014). Therefore, we hypothesize that there are likely many more loci associated with thermal tolerance in Delta Smelt, but that fell below the strict significance threshold. Accordingly, we performed a functional enrichment test on the genes linked to SNPs that passed the chromosome‐level significance threshold (62 SNPs in LD with 26 genes), as well as genes linked to SNPs in the top 0.04% of associations (547 SNPs in LD with 209 genes). This threshold was associated with a −log_10_(*p*) ≥ 3.5 (red line in Figure 3) and was chosen to encapsulate peaks of association (“skyscrapers”) of many SNPs. Genes linked with SNPs that passed the chromosome‐level significance threshold were not enriched for any GO terms. Loci passing the −log_10_(*p*) ≥ 3.5 threshold were enriched for Biological Processes GO terms including *chromatin remodeling and organization*, and *protein‐DNA complex organization*, and for Cellular Components *golgi apparatus, (intracellular) membrane‐bounded organelle, (intracellular) non‐membrane‐bounded organelle, intracellular organelle, nuclear protein‐containing complex, catalytic complex, and transferase complex* (Table S1).

**FIGURE 4:**
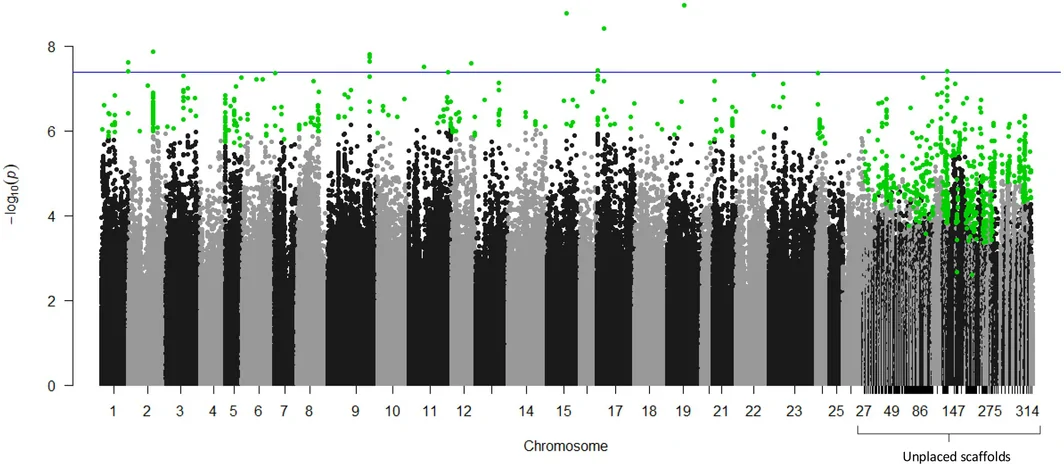
Genome‐wide association test (GWAS) for temperature‐dependent genetic effect on CTMax scores. Manhattan plot of *GEMMA* model results (−log pvalue) for GxE interactions associated with CTMax. The blue horizontal line represents the genome‐wide *p* = 0.05 significance threshold after Bonferroni correction. SNPs highlighted green are ones that pass the chromosome (or scaffold) *p* < 0.05 Bonferroni corrected significance threshold.

For the temperature‐dependent genotype effects (GxE) on CTMax, we identified 8 independent loci across 7 chromosomes that passed the genome‐wide *p*‐value threshold (Figure 4), overlapping with three genes: tufm, retreg3, and ccpg1. Functional enrichment of genes linked to SNPs that passed the chromosome‐level significance threshold (314 SNPs in LD with 150 genes, excluding SNPs found on unplaced scaffolds) was enriched for 12 Biological Processes GO terms including *(transmembrane) transport, translation, peptide metabolic process, monoatomic ion (transmembrane) transport, peptide biosynthetic process, amide metabolic process, amide biosynthetic process*, and *(establishment of) localization* (Table S2). There were 15 Molecular Function GO terms enriched, including *GTPase activator/regulator activity, (ion transmembrane) transporter activity, enzyme activator/regulator activity, channel, catalytic activity acting on RNA*, and *salt transmembrane transporter activity* (Table S2).

### 3.4 Genomic Architecture of Fork Length

**FIGURE 5:**
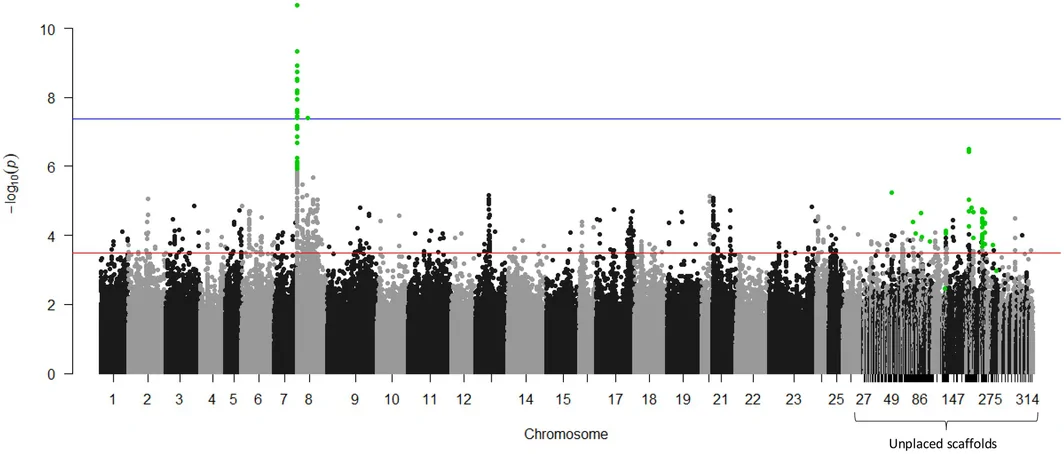
Genome‐wide association test (GWAS) for the effect of genotypic variation on fork length. Manhattan plot of *GEMMA* model results (−log *p*value) for SNPs associated with fork length. The blue horizontal line represents the genome‐wide *p* = 0.05 significance threshold after Bonferroni correction. The red horizontal line at −log *p*‐value 3.5 was chosen to encapsulate peaks of association for functional enrichment exploration. Genes linked to SNPs highlighted in green and those above the red horizontal line were tested for GO enrichment.

Using GWAS to identify loci underlying variation in fork length, we tested for the main effect of genotype on fork length (while controlling for the rearing temperature) and the temperature‐dependent genotype effect (GxE). The SNP heritability for the main genotype effect was 0.30 (SE ±0.03), with one locus of large effect on chromosome 8 (Figure 5) that was in LD with four protein coding genes: two zc2hc1a genes and two cntnap2a genes. There were many peaks of association that may contribute to variation in fork length but were of lower effect size and below statistically significant thresholds. We therefore performed a functional enrichment test on the genes linked with SNPs that passed the chromosome‐level significance threshold (71 SNPs in LD with 15 genes), as well as those linked with SNPs in the top 0.05% of associated loci (613 SNPs in LD with 192 genes). This cut‐off was associated with a −log_10_(*p*) ≥ 3.5 (red line in Figure 5) and was chosen to encapsulate stacked peaks of association (“skyscrapers”) of many SNPs. SNPs passing the chromosome‐level significance threshold were not enriched for any GO terms. Genes associated with SNPs that passed the −log_10_(*p*) ≥ 3.5 threshold were enriched for Biological Processes GO terms including *organelle organization, cellular component organization, cellular homeostasis, homeostatic process, cellular lipid metabolic process, glycerolipid process*, and *regulation of biological quality*. Enriched Molecular Processes GO terms included *protein binding* and *hydrolase activity*, and enriched Cellular Component GO terms included *receptor complex, membrane protein complex, plasma membrane protein complex*, and *plasma membrane signaling receptor complex* (Table S3).

**FIGURE 6:**
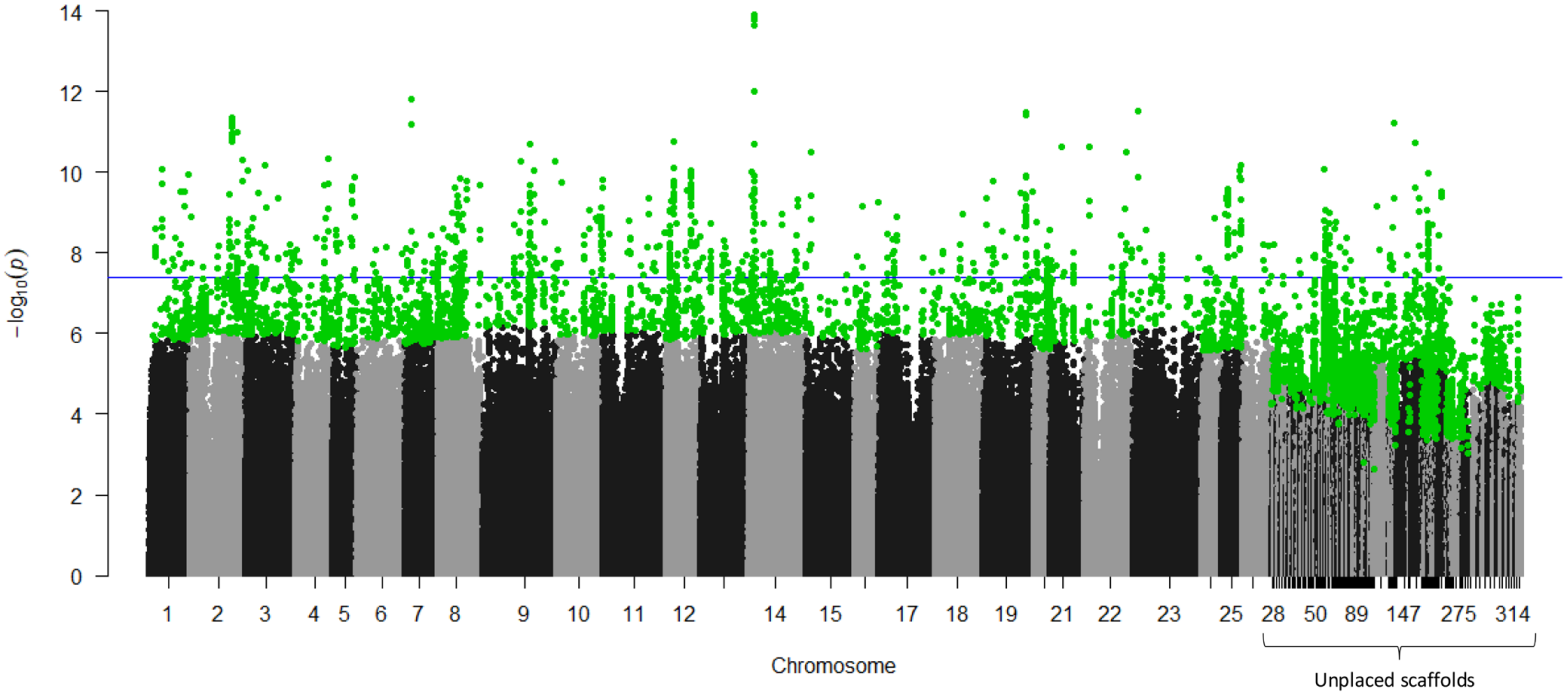
Genome‐wide association test (GWAS) for genetic effects that are temperature‐dependent on fork length. Manhattan plot of *GEMMA* model results (−log *p*value) for GxE interactions associated with fork length. The blue horizontal line represents the genome‐wide *p* = 0.05 significance threshold after Bonferroni correction. SNPs highlighted in green pass the chromosome‐level Bonferroni correction threshold. Genes linked with SNPs above the genome‐wide threshold were tested for GO enrichment.

We identified many SNPs that had a temperature‐dependent effect on fork length, suggesting that the genomic basis of body size may also depend on the rearing temperature (Figure 6). Many SNPs passed both the genome‐wide and chromosome‐level threshold. We investigated the functional enrichment of SNPs that passed the genome‐wide threshold only (828 SNPs in LD with 268 genes, excluding SNPs on the unplaced scaffolds). These SNPs were functionally enriched in Biological Processes terms, including *microtubule‐based movement, carbohydrate metabolic process*, and Molecular Function terms, including *(phosphoric ester) hydrolase activity, phosphatase activity, actin (filament) binding, (small) GTPase binding, microtubule motor activity*, and *cytoskeletal protein binding* (Table S4).

### 3.5 Signatures of Domestication Selection

**FIGURE 7:**
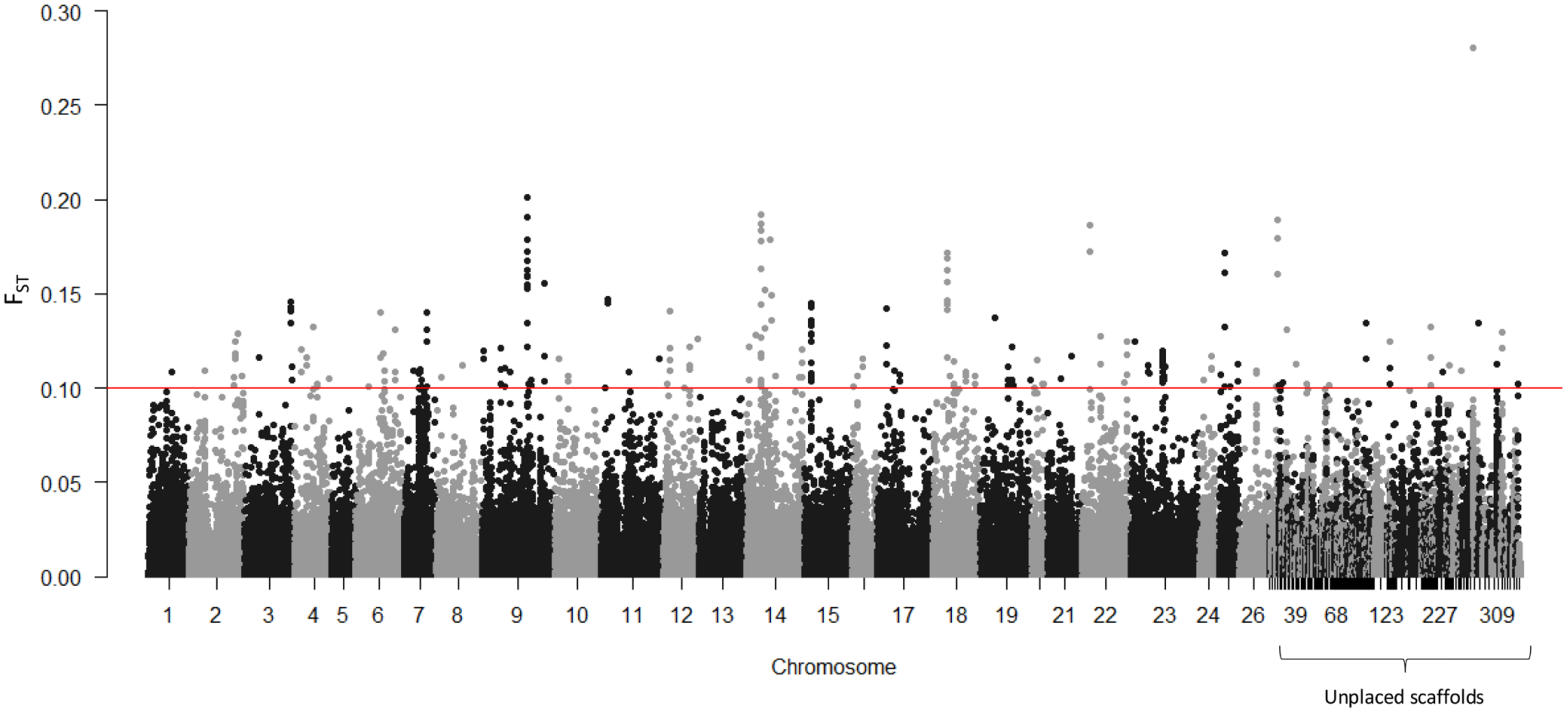
Manhattan plot of F_ST_ average across each of 10,000‐bp sliding windows between low and high domestication index (DI) adult progenitors. Genes linked with SNPs within windows above the red horizontal line (*F*_ST_ > 0.1) were tested for GO enrichment.

We detected evidence of domestication selection in the hatchery, as measured by *F*_ST_ between low and high DI groups of progenitor fish (*N* = 31 and 29, respectively). Genome‐wide average *F*_ST_ was low (0.008) but statistically significantly greater than zero (*p*‐value = 0; all permutated *F*_ST_ values were below 0.008). Additionally, some regions of the genome across almost all chromosomes displayed peaks of *F*_ST_ elevated above 0.1 (Figure 7). We performed a functional enrichment test on the SNPs in the top 0.3% of the sliding window regions (6740 SNPs in LD with 210 genes). This threshold was associated with *F*_ST_ > 0.1 (Figure 7) and was chosen to encapsulate regions of elevated divergence (“skyscrapers”). Genes linked with these high‐differentiation SNPs were enriched for Biological Processes GO terms including *chemical synaptic transmission, multicellular organism, development, anatomical structure, development, cell–cell signaling, multicellular organismal process, developmental process, anterograde trans‐synaptic signaling, trans‐synaptic signaling*, and one Molecular Function GO term *DNA‐binding transcription factor activity* (Table S5).

**FIGURE 8:**
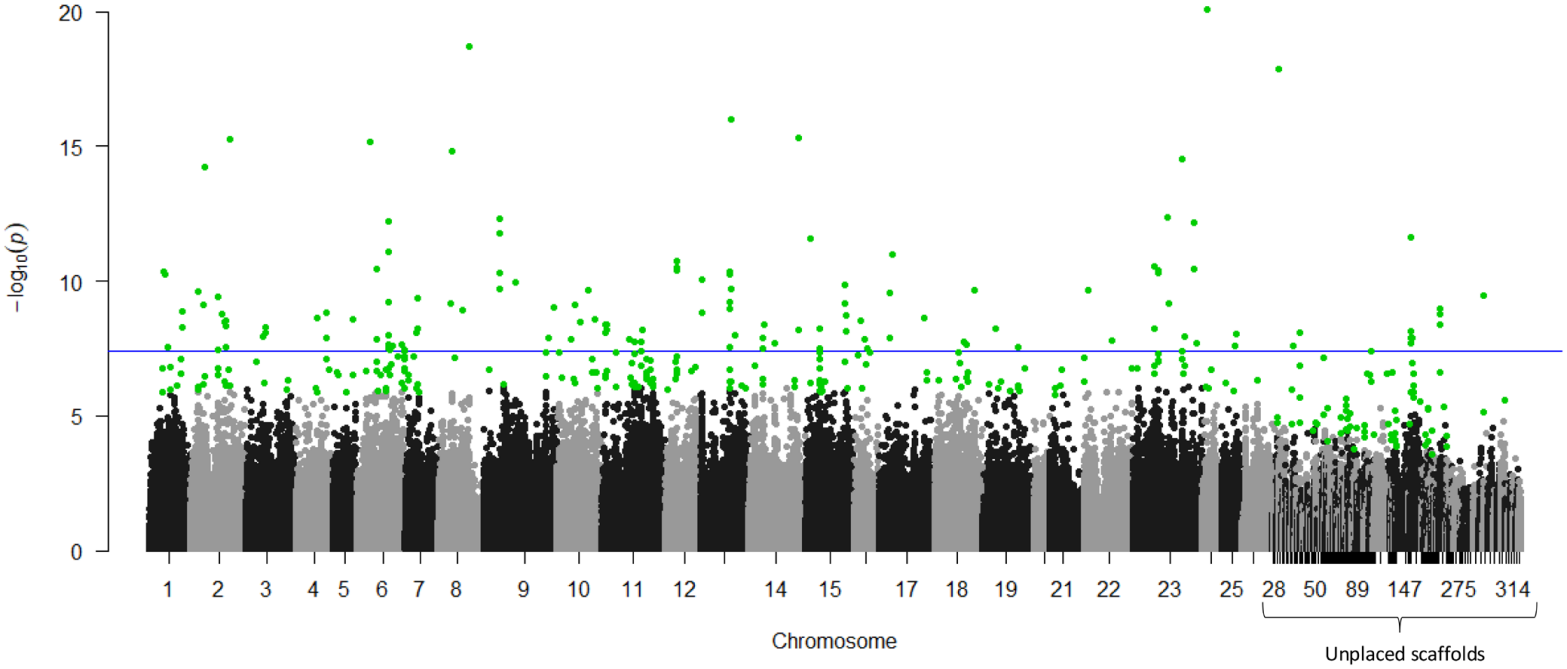
Genome‐wide association test (GWAS) for the effect of genotypic variation on domestication index (DI) in offspring. Manhattan plot of *GEMMA* model results (−log *p*value) for SNPs associated with numerical DI value. The horizontal blue line represents the genome‐wide *p* = 0.05 significance threshold after Bonferroni correction. SNPs highlighted green are those that pass the chromosome (or scaffold) *p* > 0.05 significance threshold following Bonferroni correction. Genes linked with SNPs highlighted in green and those above the blue horizontal line were tested for GO enrichment.

We also investigated loci associated with domestication (DI) in offspring using a GWAS approach that allowed us to account for relatedness among individuals. We identified many loci across multiple chromosomes associated with DI that passed the genome‐wide and chromosome‐level significance thresholds (Figure 8). We performed a functional enrichment test on genes linked with the SNPs that passed the genome‐wide significance threshold (147 SNPs in LD with 69 genes), as well as with loci that passed the chromosome‐level significance threshold (224 SNPs in LD with 165 genes, excluding SNPs on unplaced scaffolds). For those passing the genome‐wide threshold, genes were enriched for the Biological Processes GO term *vesicle‐mediated transport*, Molecular Function GO terms including (*protein‐macro) molecule adaptor activity*, and Cellular Component GO terms including *plasma membrane* and *cell periphery* (Table S6). For those passing the chromosomal‐level significance threshold, genes were enriched in Biological Processes GO terms including *(homophilic) cell adhesion* and *cell–cell adhesion*. Enrichment in Molecular Function GO terms included *protein‐macromolecule adaptor activity, phosphatidylinositol binding*, and *molecular adaptor activity*. Enrichment in Cellular Component GO terms included *cell periphery* and *plasma membrane protein complex* (Table S7).

### 3.6 Minimal Shared Genomic Basis Among Traits

Pedigree‐based animal models revealed statistically significant genetic correlations among several traits, including CTMax and fork length, CTMax and DI, and family‐level mean CTMax measured at 15°C and 18°C rearing temperatures. These correlations suggest the presence of shared genetic influences among traits. To test this hypothesis with genetic data, we evaluated overlap among genes identified in GWAS analyses for each trait and assessed SNP‐level effect sizes for both traits using the PLACO+ framework. Despite the large number of genes linked to the top 300 SNPs per GWAS (90–144 genes), there was limited overlap among analyses: traits shared only 1–6 trait‐associated loci (Table 1). However, given the large background set of possible genes (over 23,000) in LD with variant SNPs, several comparisons of overlap exceeded expectations compared to the null expectation of random association. These included overlaps that matched pedigree‐based correlations, such as between the genotype‐only associations for CTMax and: (1) its GxE associations for CTMax, (2) genotype‐only associations for fork length, and (3) low‐high progenitor F_ST_ divergence. Additionally, GxE associations for CTMax overlapped significantly with GxE associations for fork length. In contrast, SNP‐level comparisons using PLACO+ found no SNPs that passed genome‐wide nor chromosomal level thresholds between any comparisons (Figures S6–S11).

**TABLE 1:** Pairwise overlap of genes associated with each trait in GWAS analyses, including for CTMax, fork length, and domestication index (DI).

Trait (genes)	Genotype: CTMax (105)	G × E: CTMax (90)	Genotype: Fork Length (98)	G × E: Fork Length (91)	Genotype: DI (144)
Genotype: CTMax (105)	—	—	—	—	—
G × E: CTMax (90)	**6 (*p* = 2.9 × 10–6)**	—	—	—	—
Genotype: Fork Length (98)	**3 (*p* = 9.3 × 10–3)**	—	—	—	—
G × E: Fork Length (91)	—	**2 (*p* = 0.046)**	1 (*p* = 0.31)	—	—
Genotype: DI (144)	1 (*p* = 0.13)	1 (*p* = 0.42)	—	—	—
Low‐High DI FST (7)	0 (*p* = NA)	0 (*p* = NA)	0 (*p* = NA)	0 (*p* = NA)	0 (*p* = NA)
Low‐High DI F_ST_ (210)	**4 (*p* = 0.014)**	1 (*p* = 0.55)	0 (*p* = NA)	1 (*p* = 0.55)	**4 (*p* = 0.04)**

*Note:* Numbers in parentheses indicate the total number of genes that were in LD with the top 300 SNPs identified for each GWAS. For low‐high progenitor F_ST_, we compared overlap of 300 SNPs (7 genes) from the top windows, as well as SNPs above the 0.1 F_ST_ threshold (210 genes). The total number of overlapping genes between trait pairs is reported, with associated hypergeometric test *p*‐values. Bold values indicate statistically significant (*p* < 0.05) results.

### 3.7 Overlap of GWAS Candidate Genes With Transcriptomic and Epigenomic Responses

To further explore genes associated with genotype and GxE effects on CTMax and DI, we tested whether candidate genes from GWAS analyses were significantly enriched for genes that were found in Griffiths et al. (2026) to be differentially expressed or differentially methylated between DI groups or between groups reared at different temperatures. We found that most GWAS gene sets were not significantly enriched for genes that were differentially expressed or differentially methylated (Tables 2 and 3). However, the genes with genotype (GWAS) associations with DI were significantly enriched for genes that were differentially expressed between DI groups (18 genes shared between the two groups, which is more than expected by chance *p* = 0.001).

**TABLE 2:** Pairwise overlap of genes associated with variation in CTMax identified from GWAS analyses and genes differentially expressed (DEGs) or differentially methylated (DMRs) in response to rearing temperature (15°C vs. 18°C) or to rearing temperature × domestication index (DI) interactions.

	Genotype: CTMax (209)	GxE: CTMax (247)
Differentially expressed between rearing temperatures (1602)	12 (*p* = 1)	15 (*p* = 1)
Differentially expressed Temperature‐by‐DI interaction (26)	1 (*p* = 0.48)	1 (*p* = 0.54)
Differentially methylated between rearing temperatures (1244)	22 (*p* = 1)	11 (*p* = 1)

*Note:* Numbers in parentheses in the row and column headers indicate the total number of candidate genes from GWAS or the total number of DEGs/DMRs from Griffiths et al. (2026). GWAS candidate genes from genetic associations with CTMax were defined using a −log_10_(*p*) ≥ 3.5 threshold, whereas candidate genes from GxE associations with CTMax were defined using a chromosome‐level significance threshold. For each comparison, the number of overlapping genes is shown, with the associated hypergeometric *p*‐value provided in parentheses.

**TABLE 3:** Pairwise overlap of genes associated with domestication index (DI) identified from GWAS analyses or progenitor F_ST_ and genes differentially expressed (DEGs) or differentially methylated (DMRs) between low or high DI fish or to rearing temperature × domestication index (DI) interactions.

	Genotype: DI (192)	Low‐High DI F_ST_ (210)
Differentially expressed between DI groups (353)	**18 (*p* = 1.1 × 10–3)**	11 (*p* = 0.27)
Differentially expressed Temperature‐by‐DI interaction (26)	1 (*p* = 0.45)	0
Differentially methylated between DI groups (965)	14 (*p* = 1)	25 (*p* = 1)

*Note:* Numbers in parentheses in the row and column headers indicate the total number of candidate genes from GWAS or the total number of DEGs/DMRs from Griffiths et al. (2026). GWAS candidate genes from the genetic association with DI were defined using a chromosome‐level significance threshold, whereas candidate genes for F_ST_ were based on values above 0.1. For each comparison, the number of overlapping genes is shown, with the associated hypergeometric *p*‐value provided in parentheses. Bold value indicate statistically significant (*p* < 0.05) results.

## 4 Discussion

**FIGURE 9:**
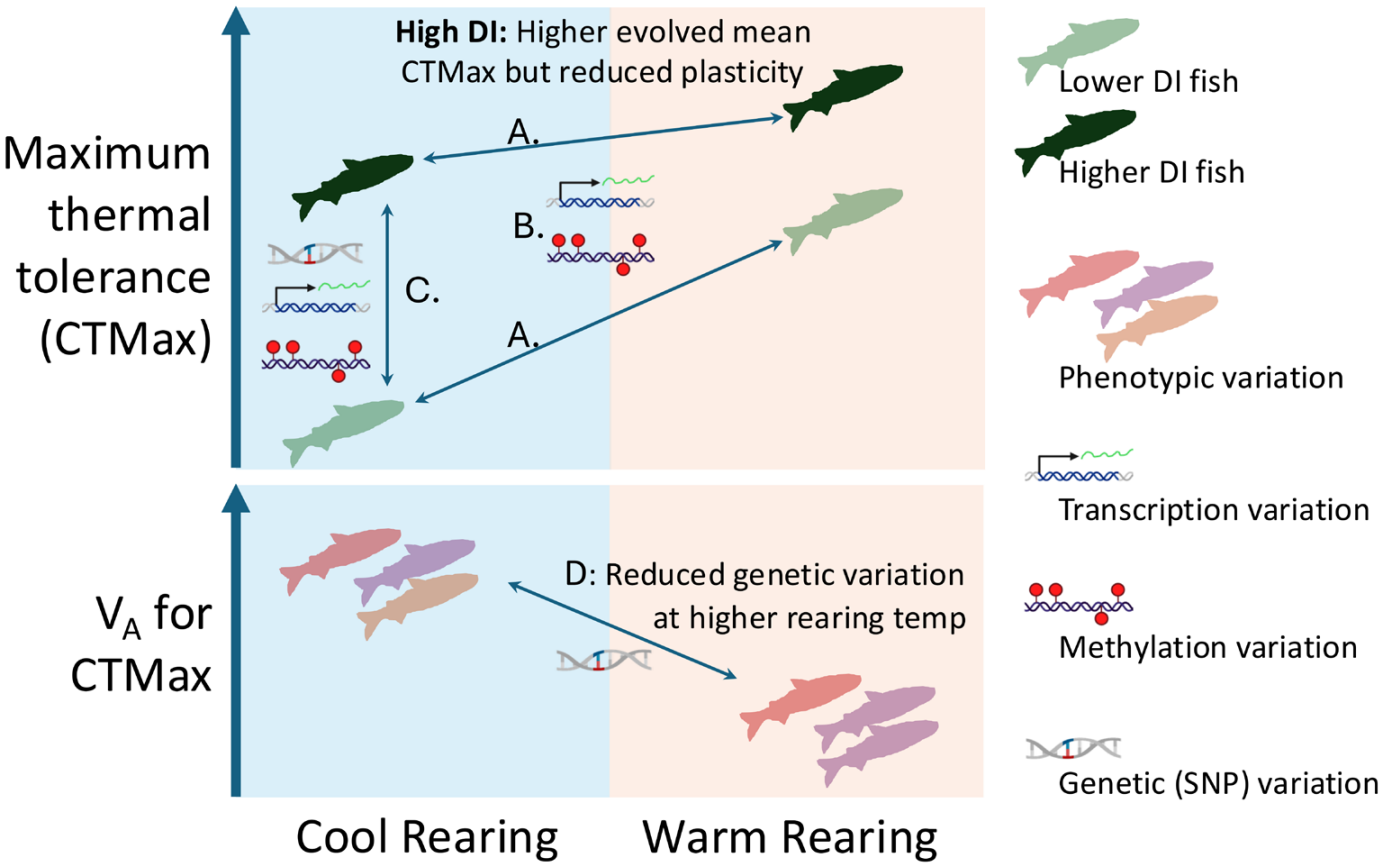
Conceptual summary of main findings from this study and Griffiths et al. (2026). (A) Delta Smelt can increase their thermal tolerance (CTMax) if reared at elevated temperature (acclimation or phenotypic plasticity). While higher domestication index (DI; black) fish have higher mean thermal tolerance at both rearing temperatures, they have reduced thermal plasticity compared to lower DI (green) fish (slope connecting lower DI fish between cool and warm rearing is steeper than for the slope connecting higher DI fish). (B) Rearing at different temperatures induced transcriptomic and methylomic changes but with similar responses among low and high DI fish (see Griffiths et al. 2026). (C) Phenotypic variation in CTMax that distinguishes low from high DI fish is likely underpinned by evolved genetic variation that manifest as transcriptomic and methylomic variation. (D) Additive genetic variation in thermal resilience is reduced in warmer rearing environments.

As captive‐reared individuals are released into the wild they may face altered environments or a legacy of domestication selection that may cause traits to be mismatched to contemporary conditions. In the Delta Smelt hatchery, recent cohorts have relied on captive‐born broodstock due to the scarcity of wild individuals and captive rearing temperatures are stable and cool during the summer. However, upon release, captive Delta Smelt will be introduced into a habitat that has been rapidly warming, where critical thermal thresholds dictate survival (Davis et al. 2024). Here, we assessed whether captive Delta Smelt harbor the genetic variation necessary to adapt to elevated temperatures, and how rearing temperature, body size, and domestication index influence this genetic infrastructure. We discovered some thermal acclimation ability (plasticity) during early life rearing at elevated temperature, but this was accompanied by a reduced plastic response in more highly domesticated Delta Smelt. Importantly, our companion study (Griffiths et al. 2026) found that both low and high DI fish still share similar molecular responses (transcriptomic and methylomic) to rearing temperatures, suggesting that the genomic mechanisms regulating thermal acclimation have not diverged. Genetic variation for thermal tolerance was reduced under warmer temperatures, signaling a constraint on adaptation potential. While few loci significantly contributed to the genomic architecture of thermal tolerance across both rearing temperatures, we identified extensive loci with significant rearing temperature‐dependent effects, indicating that the genomic architecture is instead highly contingent on acclimation conditions. Furthermore, we demonstrate the influence of domestication selection; between low and high DI fish we detected loci with large allele frequency differences, as well as divergence in their transcriptomes and methylomes (Griffiths et al. 2026). However, we found minimal overlap between loci associated with domestication and thermal tolerance, suggesting that these two traits possess separate genetic underpinnings. We summarize these findings, coupled with those reported in the companion study Griffiths et al. (2026) in a conceptual model (Figure 9).

### 4.1 Drivers of Thermal Tolerance and Adaptive Potential in Delta Smelt

The ability to increase upper temperature tolerance via acclimation is an important physiological mechanism for maintaining fitness during extreme heat events. Consistent with previous findings (Griffiths et al. 2026), Delta Smelt showed a modest yet statistically significant capacity for thermal plasticity (acclimation), where fish reared at 18°C exhibited an increase in CTMax (0.6°C) compared to those reared at 15°C (Figure 1). The observed upper limit of CTMax (~30°C–31°C) was consistent across both rearing temperatures and previous studies (Komoroske et al. 2014), and likely represents a physiological barrier for the species (Pörtner et al. 2017). Although this threshold (30°C–31°C) exceeds temperatures that caused total mortality of adults during experimental releases (26°C), it is widely acknowledged that CTMax methodology does not always accurately reflect sustained exposure to extreme heat (Bartlett et al. 2022; Brauner and Richards 2020). Additionally, larval Delta Smelt are reported to have higher upper thermal tolerance limits than adults (Komoroske et al. 2014).

Domestication also altered thermal physiology within Delta Smelt, as more highly domesticated fish (high DI) exhibited higher CTMax than lower DI fish at both rearing temperatures. There was a 0.24°C increase in CTMax with each 1‐unit increase of DI (each DI unit represents 1 generation spent in the hatchery). This suggests that genetic variation for thermal tolerance may be partitioning among fish with different degrees of hatchery ancestry. However, directional thermal selection in captivity is unlikely to have caused the observed increased thermal tolerance, given that Delta Smelt are reared at cooler temperatures than what Delta Smelt experience in the wild. Instead, thermal tolerance may be genetically correlated to other traits under selection in the hatchery (e.g., metabolic rates, feeding capacity, stress tolerance).

To investigate the relative contributions of the rearing environment and genetic variation on CTMax variation, we estimated the heritability of the trait. We observed moderate heritabilities for CTMax (0.15 and 0.26 pedigree‐based, and 0.14 genomics based), which suggests that while variation in thermal tolerance has a large environmentally dependent component, there is a significant but modest genetic basis that can be acted upon by selection. The heritability of CTMax measured in Delta Smelt is comparable to or lower than thermal tolerance heritabilities estimated in other species such as Atlantic salmon (0.20 and 0.47) and rainbow trout (0.41) (Benfey et al. 2024; Gonen et al. 2024; Perry et al. 2005).

As the currency of evolutionary change, genetic diversity is important for population persistence during adjustment to environmental shifts; consequently, a loss of variation—particularly at quantitative trait loci—can compromise evolutionary potential. Despite similar heritabilities for thermal tolerance between fish reared at the two rearing temperatures, additive genetic variation and evolvability were reduced at the warmer temperature (18°C). Phenotypic variation was also reduced in the warmer temperature (Figure 1A), corresponding with reduced residual and environmental variation in the animal model (Table S1). A reduction in both additive genetic variance and total phenotypic variance at the warmer rearing temperature explains the similar heritability estimates between the two temperatures. If CTMax was under continuous directional selection, our estimates of evolvability correspond to a 0.25% and 0.06% change in CTMax per generation, at rearing temperatures 15°C and 18°C, respectively. This suggests that while Delta Smelt possesses standing genetic variation to modify thermal tolerance, this capacity will be constrained under warmer conditions. A reduction in CTMax variability and heritability at elevated rearing temperatures has been documented in other fish species, such as Atlantic salmon and killifish (Debes et al. 2021; Rost‐Komiya et al. 2026). Taken together, these findings suggest that the increased physiological stress and reduced heritability of thermal tolerance that we observe at elevated temperatures may be a shared phenomenon across fishes, which will have broad implications for our understanding of the extent to which fish may be able to cope with climate change.

We detected evidence for genetic variation for thermal tolerance plasticity (genetic associations with CTMax that varied depending on rearing temperature), suggesting genotypes differ in their capacity to acclimate to warmer environments. Our previous work demonstrated that Delta Smelt that were more highly domesticated had reduced capacity for thermal acclimation (reduced phenotypic plasticity) compared to less domesticated fish (Griffiths et al. 2026). Stable rearing temperatures in the Delta Smelt hatchery may have, over generations, contributed to a loss of thermal plasticity in more highly domesticated fish. Despite this, we were unable to detect differences in additive genetic variation and heritability for CTMax between low, medium, and high DI fish. Although higher domesticated fish may have lost genetic variation for thermal plasticity, they still maintain the ability to evolve a higher mean thermal tolerance.

### 4.2 Genomic Architecture of Thermal Tolerance

Given evidence of thermal tolerance heritability in Delta Smelt, we used GWAS to reveal the number and identity of associated genetic loci. We found only a few SNPs that passed statistical significance thresholds for association with thermal tolerance across both rearing temperatures, suggesting variance in this trait is most likely polygenic—including many loci of small effect. Identifying the specific loci that could be targets of selection for thermal tolerance in fish remains a challenging task (Gonen et al. 2024; Ignatz et al. 2025). Heat tolerance has been demonstrated to be a highly polygenic trait in other species (Fuller et al. 2020; Gonen et al. 2024; Griffiths et al. 2020; Michalak et al. 2019; Tobler et al. 2014). SNPs associated with polygenic traits are difficult to detect, since the effect size of each contributing locus is small (Yeaman 2015). Since the trait is likely polygenic, we sought to test whether a wider set of SNPs with individually lower association values might also implicate mechanisms underlying variation in thermal physiology. Indeed, tests for functional enrichment of the top 0.04% of the SNPs identified many more functions and cellular components, which were consistent with other studies that have shown that physiological and cellular heat stress has been characterized by loss of ionic and osmotic balance (reviewed by Evans 2008) and the compromise of lipid membrane fluidity (Hochachka and Somero 2002).

While we detected a relatively limited number of loci with consistent effects on CTMax across rearing temperatures, we identified a large number of loci where the genetic association with CTMax depended on the rearing temperature. This environmental dependence (rearing temperature) of genotype on phenotype is consistent with our finding that additive genetic variation for CTMax differed between the two rearing temperatures. These temperature‐dependent loci were enriched for a wide‐range of functional categories including gene regulation and structure and cellular organization (i.e., chromatin organization, protein‐DNA organization, and cellular component organization), suggesting that loci with a GxE effect may involve structural changes to the genome and rearrangement of cellular structures. These loci may also cause adjustments to metabolic processes (i.e., ATP‐dependent activity) and cell signaling and communication (i.e., cell–cell signaling, endopeptidase inhibitor activity, and enzyme regulator activity).

In a companion study, we demonstrated that elevated rearing temperatures resulted in large transcriptomic and methylomic changes in juvenile Delta Smelt (Griffiths et al. 2026). However, SNPs associated with CTMax and with GxE CTMax were not linked to the same genes as those involved in temperature‐induced transcriptional and epigenetic changes. Therefore, the segregating genetic variation associated with thermal tolerance limits (i.e., CTMax) is in different molecular machinery than that governing physiological acclimation during early life development. While rearing at 18°C generally primed fish to develop higher CTMax values, the GWAS and DEG/DMR methodology were measuring two related, but slightly different traits: acute upper thermal tolerance limits (CTMax) and long‐term acclimation to elevated temperatures.

### 4.3 Fork Length, Covariates, and Genomic Architecture

Fork length in Delta Smelt is a complex trait influenced by both rearing temperature and genetic variation. Fork length showed a positive plastic response to rearing temperature, where larger fish emerged at higher rearing temperature (Figure 1C). Warm rearing temperatures often increase metabolic rates, which can lead to faster growth (Wood and McDonald 1997). However, we also estimated large pedigree‐ and genomic‐based heritabilities for this trait (0.58 and 0.50 pedigree‐based, 0.30 genomic‐based), consistent with genetic variation contributing to trait variation. Indeed, we detected one large‐effect locus that overlapped with four genes, two of which are annotated, but with functions not typically associated with size or growth. An additional signal of subtle polygenic variation associated with this trait was distributed across multiple chromosomes. Functional enrichment of genes in these peaks suggests that cellular structure and organization, metabolism, homeostasis, and cell signaling contribute to variation in fish length. Compared to the number of loci associated with genetic variation for fork length, there were many more loci that had a rearing temperature dependent effect on fork length. These GxE loci for fork length were enriched for cellular structure and metabolism functions, as well as protein binding and regulation (such as GTPase binding, which acts as a molecular switch known to control a variety of functions, including growth; (Satoh 2020)).

Fork length displayed strong negative phenotypic and genetic correlations with CTMax, indicating that larger Delta Smelt have reduced thermal tolerance. Similar findings have also been found in other salmon species (Bartlett et al. 2022; Benfey et al. 2024; Debes et al. 2021). Our observed negative genetic correlation between these two traits suggests a potential shared genetic basis, with the implication that selection may be constrained from simultaneously increasing both fork length and CTMax. Although this trade‐off could be a concern for fish in a warming climate, we found that this effect was diminished at warmer rearing temperatures. This may be a result of reduced genetic variation for CTMax at 18°C, and phenotypic plasticity may play a larger role in determining the final phenotype. At the genomic level, we found some significant overlap in genes for CTMax and fork length GWAS (Table 1), which is consistent with at least some shared genetic basis.

### 4.4 Signatures of Domestication Selection

Despite careful genetic management, evidence for domestication in the Delta Smelt hatchery has been observed for a variety of traits, such as reproductive success, growth, and survival. In the hatchery, Delta Smelt spawners that have greater hatchery ancestry tend to produce more offspring that survive to reproductive age compared to fish with shallower hatchery ancestry (Finger et al. 2018; LaCava et al. 2023). However, in this study, we found that larval (71–79 dpf) survival rates among families were similar across rearing temperatures and DI categories, and that DI had only a small effect on size. In contrast, previous work found pronounced differences in size and survival among low and high DI fish (Chase et al. 2024; Ellison et al. 2023), but those studies included very low DI fish that were not available for our experiments.

We identified genomic divergence within both the progenitor and offspring populations. We detected elevated F_ST_ differentiation between low and high DI progenitors as well as SNPs associated with continuous variation in DI within the offspring population. These results may represent an underlying genomic basis for divergence in traits within hatchery populations (Finger et al. 2018; Griffiths et al. 2026; LaCava et al. 2023). Divergent loci were functionally enriched in categories related to development and morphogenesis (i.e., multicellular organism development, anatomical structural development, and developmental processes), which suggests selection on development and growth traits. These loci may contribute to the divergence in physiology and morphology that we observed between low and high DI fish, but may also influence other morphological or developmental traits that we did not measure (Figure 1C). For example, loci distinguishing low from high DI fish were linked to genes that were functionally enriched for GO categories related to synaptic and neural signaling (i.e., synaptic signaling, cell–cell signaling, and chemical synaptic transmission), which suggests that domestication has altered neurological and behavioral systems as observed in other domesticated fish (e.g., (Ghazal et al. 2025)).

Genomic variation distinguishing low from high DI fish may be associated with divergence in gene expression between low and high DI fish. We detected significant overlap in the gene set identified by GWAS association with DI and the gene set that was differentially expressed between low and high domesticated fish from Griffiths et al. (2026). Our results are consistent with domestication selection being a complex process that influences both genetic variation and molecular response pathways. Accordingly, one may consider the influence of hatchery conditions not only on survival but also as a filter for genotypic variation and as a lever that influences the evolutionary forces that govern the fate of that variation.

## 5 Conclusion and Hatchery Implications

Building resilience to environmental variation should be a goal of most conservation hatcheries that release captive animals back into environments altered by climate change (Bradford et al. 2025; Fraser 2008). Maintaining or enriching for thermal tolerance will be critical for the success of the Delta Smelt supplementation program, especially as recent experimental releases have experienced high temperature‐driven mortality (Baerwald et al. 2023; Davis et al. 2024; U.S. Fish and Wildlife Service 2020). Our results indicate the existence of standing genetic variation which could be the substrate for natural selection to adaptively increase thermal tolerance. However, this adaptive capacity may be constrained under warmer conditions due to the observed reduction in heritability of thermal tolerance at higher rearing temperatures. Consequently, the potential for adaptation to current temperature conditions in the wild may be limited given that wild Delta Smelt are already experiencing summer temperatures above those used in experiments presented here (Davis et al. 2024). Delta Smelt also possess the capacity for improving thermal tolerance through acclimation, and the presence of many loci with rearing temperature‐dependent impacts on thermal tolerance suggests there is genetic variation for thermal plasticity. However, selection in captivity may have reduced thermal plasticity. Thermal performance traits in Delta Smelt are therefore a combination of genetic variation, genotype‐by‐environment interactions, and acclimation, making it challenging to predict the interventions that will maximize the success of supplementation. Simple marker‐assisted breeding will be challenging, given the highly polygenic nature of thermal tolerance and the potential for complex interactions with other genetic variation important for fitness in the wild. Given that all Delta Smelt now originate from the hatchery, tracking phenotypic traits and domestication indices following supplemental releases will be important for determining how these factors influence fitness in the wild.

## Funding

This research was supported by the California Department of Fish and Wildlife (Prop 1: Watershed Restoration Grant Program/Delta Water Quality and Ecosystem Restoration Program. Grant Q1996041, PIs Dr. Andrew Whitehead, Dr. Nann Fangue, Dr. Tien‐Chieh Hung). Additional funding provided by the UC Agricultural Experiment Station (2098‐H) to N.A.F.

## Conflicts of Interest

The authors declare no conflicts of interest.

## Supporting information


**Figure S1:** Factorial breeding block design and offspring rearing temperature experimental design. Families were generated from adult smelt of low, medium, or high DI category. Each block consisted of 5 males (numbers) crossed with 2 females (letters) from the same DI category. Each family was designated by the number and letter combo of its parents (i.e., C10). There were 4 blocks for the low DI group, 3 blocks for the medium DI group, 3 blocks for the high DI group, and 3 blocks which included a “mix” of low, medium, and high DI fish. There were a total of 88 parents crossed and 130 families generated. Each family was evenly split into four groups and reared at either 15C or 18C (with replicates for each temperature). Fish from the same DI category (low, medium, high or mix) were kept together in a single tank, with four tanks fed by a single header tank for each temperature and replicate (i.e., Closed recirculating “system”).
**Figure S2:** Box plot distribution of each individual offspring's numerical domestication index (DI) number in each low and high DI category. In FCCL, low DI fish are defined as having a numerical DI below 7, medium between 7 and 10, and high DI fish above 10. Box plot displays median (middle line of box), the first and third quartile of data (box edges), and full range of data (whiskers). Mean numerical DI in the low group was 6.06, medium group was 8.56, and the high group was 10.96.
**Figure S3:** The r2 correlation between imputed genotypes of a down‐sampled individuals to 1× coverage (in minor allele frequency bins) and highly‐confident genotype calls from the same individual at 30× coverage. Higher correlations suggest that the imputation methods perform more accurately at higher minor allele frequencies in the sampled population reference panel. We filtered our final reference panel to a minor allele frequency cut‐off of 2%, resulting in an imputation accuracy of 80%–85%.
**Figure S4:** (A) Percent recovered offspring per family reared at either 15°C (blue) or 18°C (orange). Each family is designated by its numerical domestication index (DI). Low DI < 7, medium DI 7–10, high DI > 10. (B) Plasticity of survival (Percent recovered offspring per family) at two different rearing temperatures, 15C and 18C.
**Figure S5:** Percent recovered offspring per family by the mean family CTMax reared at either 15°C (blue) or 18°C (orange).
**Figure S6:** Manhattan plot of PLACO+ results (−log *p*value) for SNPs associated with both genotype effects on CTMax and temperature‐dependent genotype effects on CTMax. No SNPs passed the genome‐wide 0.05 significance threshold after Bonferroni correction.
**Figure S7:** Manhattan plot of PLACO+ results (−log *p*value) for SNPs associated with both genotype effects on CTMax and genotype effects on DI. No SNPs passed the genome‐wide 0.05 significance threshold after Bonferroni correction.
**Figure S8:** Manhattan plot of PLACO+ results (−log *p*value) for SNPs associated with both genotype effects on fork length and temperature‐dependent genotype effects on fork length. No SNPs passed the genome‐wide 0.05 significance threshold after Bonferroni correction.
**Figure S9:** Manhattan plot of PLACO+ results (−log *p*value) for SNPs associated with both genotype effects on CTMax and genotype effects on fork length. No SNPs passed the genome‐wide 0.05 significance threshold after Bonferroni correction.
**Figure S10:** Manhattan plot of PLACO+ results (−log *p*value) for SNPs associated with both temperature‐dependent genotype effects on CTMax and temperature‐dependent genotype effects on fork length. No SNPs passed the genome‐wide 0.05 significance threshold after Bonferroni correction.
**Figure S11:** Manhattan plot of PLACO+ results (−log *p*value) for SNPs associated with both temperature‐dependent genotype effects on CTMax and genotype effects on DI. No SNPs passed the genome‐wide 0.05 significance threshold after Bonferroni correction.

**Table S1:** GO functional enrichment for SNPs associated with CTMax in the top 0.04% of GEMMA model results (547 SNPs that were in LD with 209 unique genes). This cut‐off was associated with a −log *p*‐value of 3.5 (Figure 2) and was chosen to encapsulate stacked peaks (“skyscrapers”) of many SNPs.
**Table S2:** GO functional enrichment for SNPs associated with a GxE interaction for CTMax that passed the chromosome‐level significance threshold (965 SNPs that are in LD with 247 unique genes).
**Table S3:** GO functional enrichment for SNPs associated with fork length in the top 0.05% of GEMMA model results. This cut‐off was associated with a −log *p*‐value of 3.5 (Figure 4) and was chosen to encapsulate stacked peaks (“skyscrapers”) of many SNPs.
**Table S4:** GO functional enrichment for SNPs associated with a GxE interaction for fork length that passed the chromosome‐level significance threshold (828 SNPs in LD with 268 unique genes).
**Table S5:** GO functional enrichment for SNPs that showed elevated peaks of divergence between low and high DI parent fish. These SNPs are in the top 0.3% of the sliding window regions (6740 SNPs that are in LD with 210 unique genes). This cut‐off was associated with a Fst above 0.1 (Figure 8) and was chosen to encapsulate stacked peaks (“skyscrapers”) of many SNPs.
**Table S6:** GO functional enrichment for SNPs associated with domestication index (DI) in offspring fish that passed the genome‐wide significance threshold (147 SNPs that are in LD with 69 unique genes).
**Table S7:** GO functional enrichment for SNPs associated with domestication index (DI) in offspring fish that passed the chromosome‐level significance threshold (403 SNPs that are in LD with 192 unique genes).

## Data Availability

Sequencing data are deposited in the National Center for Biotechnology Information's Short Reads Archive (BioProject PRJNA1152625). Scripts and code for physiological and genomic analyses are available on GitHub: https://github.com/JoannaGriffiths/DeltaSmelt_Genetics. The progenitor reference panel can be found on Dryad (DOI: https://doi.org/10.5061/dryad.qz612jmx7).

## References

[eva70317-bib-0001] Alexa, A. , and J. Rahnenführer . 2007. “Gene Set Enrichment Analysis With topGO.” Bioconductor Improvements 27: 776.

[eva70317-bib-0002] Ali, O. A. , S. M. O'Rourke , S. J. Amish , et al. 2016. “Rad Capture (Rapture): Flexible and Efficient Sequence‐Based Genotyping.” Genetics 202, no. 2: 389–400. 10.1534/genetics.115.183665. 26715661 PMC4788223

[eva70317-bib-0003] Baerwald, M. R. , N. Kwan , C. Pien , et al. 2023. “Captive‐Reared Delta Smelt ( *Hypomesus transpacificus* ) Exhibit High Survival in Natural Conditions Using In Situ Enclosures.” PLoS One 18, no. 5: e0286027. 10.1371/journal.pone.0286027. 37235546 PMC10218733

[eva70317-bib-0004] Bartlett, C. B. , A. F. Garber , S. Gonen , and T. J. Benfey . 2022. “Acute Critical Thermal Maximum Does Not Predict Chronic Incremental Thermal Maximum in Atlantic Salmon ( *Salmo salar* ).” Comparative Biochemistry and Physiology Part A: Molecular & Integrative Physiology 266: 111143. 10.1016/j.cbpa.2022.111143. 34995773

[eva70317-bib-0005] Bates, D. , M. Maechler , B. Bolker , and S. Walker . 2014. “Lme4: Linear Mixed‐Effects Models Using Eigen and S4. R Package Version.” (pp. 1–7).

[eva70317-bib-0006] Beitinger, T. L. , W. A. Bennett , and R. W. Mccauley . 2000. “Temperature Tolerances of North American Freshwater Fishes Exposed to Dynamic Changes in Temperature.” Environmental Biology of Fishes 58: 237–275.

[eva70317-bib-0007] Benfey, T. J. , S. Gonen , C. B. Bartlett , and A. F. Garber . 2024. “Thermal Tolerance Has High Heritability in Atlantic Salmon, *Salmo salar* .” Aquaculture Reports 37: 102249. 10.1016/j.aqrep.2024.102249.

[eva70317-bib-0008] Bradford, M. J. , L. E. Kwong , C. A. Holt , B. C. Ramshaw , and R. V. Galbraith . 2025. “A Framework for the Use of Conservation Hatcheries to Support Wild Pacific Salmon Recovery in Canada.” Facets 10: 1–19. 10.1139/facets-2024-0240.

[eva70317-bib-0009] Bradshaw, A. D. 1965. “Evolutionary Significance of Phenotypic Plasticity in Plants.” In Advances in Genetics, edited by E. W. Caspari and J. M. Thoday , vol. 13, 115–155. Academic Press. 10.1016/S0065-2660(08)60048-6.

[eva70317-bib-0010] Brauner, C. J. , and J. G. Richards . 2020. “Physiological Performance in Aquaculture: Using Physiology to Help Define Optimal Conditions for Growth and Environmental Tolerance.” In Aquaculture, vol. 38, 83–121. Elsevier. 10.1016/bs.fp.2020.10.001.

[eva70317-bib-0011] California Natural Diversity Database (CNDDB) . 2024. State and Federally Listed Endangered and Threatended Animal of California. California Department of Fish and Wildlife.

[eva70317-bib-0012] Chase, S. N. , B. E. Davis , E. W. Carson , et al. 2024. “Evaluating the Impact of Captive Ancestry on the Growth and Survival of Delta Smelt in a Captive Environment.” Aquaculture Reports 36: 102169. 10.1016/j.aqrep.2024.102169.

[eva70317-bib-0013] Danecek, P. , A. Auton , G. Abecasis , et al. 2011. “The Variant Call Format and VCFtools.” Bioinformatics 27, no. 15: 2156–2158. 10.1093/bioinformatics/btr330. 21653522 PMC3137218

[eva70317-bib-0014] Danecek, P. , J. K. Bonfield , J. Liddle , et al. 2021. “Twelve Years of SAMtools and BCFtools.” GigaScience 10, no. 2: giab008. 10.1093/gigascience/giab008. 33590861 PMC7931819

[eva70317-bib-0015] Davis, B. E. , D. E. Cocherell , T. Sommer , et al. 2019. “Sensitivities of an Endemic, Endangered California Smelt and Two Non‐Native Fishes to Serial Increases in Temperature and Salinity: Implications for Shifting Community Structure With Climate Change.” Conservation Physiology 7, no. 1: 1–16. 10.1093/conphys/coy076. PMC6387996 30842886

[eva70317-bib-0016] Davis, B. E. , B. G. Hammock , N. Kwan , et al. 2024. “Insights From a Year of Field Deployments Inform the Conservation of an Endangered Estuarine Fish.” Conservation Physiology 12, no. 1: coae088. 10.1093/conphys/coae088. 39726938 PMC11669484

[eva70317-bib-0017] Debes, P. V. , M. F. Solberg , I. H. Matre , L. Dyrhovden , and K. A. Glover . 2021. “Genetic Variation for Upper Thermal Tolerance Diminishes Within and Between Populations With Increasing Acclimation Temperature in Atlantic Salmon.” Heredity 127, no. 5: 455–466. 10.1038/s41437-021-00469-y. 34446857 PMC8551234

[eva70317-bib-0018] Durinck, S. , Y. Moreau , A. Kasprzyk , et al. 2005. “BioMart and Bioconductor: A Powerful Link Between Biological Databases and Microarray Data Analysis.” Bioinformatics 21, no. 16: 3439–3440. 10.1093/bioinformatics/bti525. 16082012

[eva70317-bib-0019] Durinck, S. , P. T. Spellman , E. Birney , and W. Huber . 2009. “Mapping Identifiers for the Integration of Genomic Datasets With the R/ Bioconductor Package biomaRt.” Nature Protocols 4, no. 8: 1184–1191. 10.1038/nprot.2009.97. 19617889 PMC3159387

[eva70317-bib-0020] Ellison, L. , M. M. Rahman , A. J. Finger , et al. 2023. “Size, Fecundity and Condition Factor Changes in Endangered Delta Smelt *Hypomesus transpacificus* Over 10 Generations in Captivity.” Aquaculture, Fish and Fisheries 3, no. 4: 353–365. 10.1002/aff2.116.

[eva70317-bib-0021] Etterson, J. R. , and R. G. Shaw . 2001. “Constraint to Adaptive Evolution in Response to Global Warming.” Science 151, no. 5540: 151–154. 10.1126/science.1063656. 11588260

[eva70317-bib-0080] Evans, D. 2008. “Teleost Fish Osmoregulation: What Have we learned Since August Krogh, Homer Smith, and Ancel Keys.” American Journal of Physiology Regulatory Integrative and Comparative Physiology 295: R704–R713. 18525009 10.1152/ajpregu.90337.2008

[eva70317-bib-0022] Finger, A. J. , B. Mahardja , K. M. Fisch , et al. 2018. “A Conservation Hatchery Population of Delta Smelt Shows Evidence of Genetic Adaptation to Captivity After 9 Generations.” Journal of Heredity 109: 689–699. 10.1093/jhered/esy035. 30016452

[eva70317-bib-0023] Fiumera, A. C. , B. A. Porter , G. Looney , M. A. Asmussen , and J. C. Avise . 2004. “Maximizing Offspring Production While Maintaining Genetic Diversity in Supplemental Breeding Programs of Highly Fecund Managed Species.” Conservation Biology 18, no. 1: 94–101. 10.1111/j.1523-1739.2004.00521.x.

[eva70317-bib-0024] Fox, J. , and S. Weisberg . 2019. An R Companion to Applied Regression. Sage. https://socialsciences.mcmaster.ca/jfox/Books/Companion/.

[eva70317-bib-0025] Frankham, R. 2010. “Challenges and Opportunities of Genetic Approaches to Biological Conservation.” Biological Conservation 143, no. 9: 1919–1927. 10.1016/j.biocon.2010.05.011.

[eva70317-bib-0026] Fraser, D. J. 2008. “How Well Can Captive Breeding Programs Conserve Biodiversity? A Review of Salmonids.” Evolutionary Applications 1, no. 4: 535–586. 10.1111/j.1752-4571.2008.00036.x. 25567798 PMC3352391

[eva70317-bib-0027] Fuller, Z. L. , V. J. L. Mocellin , L. A. Morris , et al. 2020. “Population Genetics of the Coral *Acropora millepora* : Toward Genomic Prediction of Bleaching.” Science 369, no. 6501: eaba4674. 10.1126/science.aba4674. 32675347

[eva70317-bib-0028] Ghazal, A. , R. Paul , A. S. Tarkan , et al. 2025. “Domestication as the Driver of Lower Chronic Stress Levels in Fish in Catch‐and‐Release Recreational Fisheries and Aquaculture Versus Wild Conspecifics.” PLoS One 20, no. 6: e0326497. 10.1371/journal.pone.0326497. 40560983 PMC12193907

[eva70317-bib-0029] Gonen, S. , T. J. Benfey , and A. F. Garber . 2024. “The Genomic Architecture of High Temperature Tolerance in a Year Class of Atlantic Salmon.” Aquaculture 578: 740020. 10.1016/j.aquaculture.2023.740020.

[eva70317-bib-0030] Griffiths, J. S. , A. J. Finger , M. R. Baerwald , et al. 2026. “Highly and Lowly Domesticated Endangered Fish From a Conservation Hatchery Diverge in Their Thermal Physiology, Transcriptome, and Methylome.” Evolutionary Applications 19, no. 3: e70220. 10.1111/eva.70220. 42015957 PMC13093269

[eva70317-bib-0031] Griffiths, J. S. , Y. Kawji , and M. W. Kelly . 2020. “An Experimental Test of Adaptive Introgression in Locally Adapted Populations of Splash Pool Copepods.” Molecular Biology and Evolution 38, no. 4: 1306–1316. 10.1093/molbev/msaa289. PMC8042754 33306808

[eva70317-bib-0032] Hadfield, J. D. 2010. “MCMCglmm: MCMC Methods for Multi‐Response GLMMs in R.” Journal of Statistical Software 33, no. 2: 1–22. 10.1002/ana.22635.20808728

[eva70317-bib-0033] Halverson, G. H. , C. M. Lee , E. L. Hestir , et al. 2022. “Decline in Thermal Habitat Conditions for the Endangered Delta Smelt as Seen From Landsat Satellites (1985–2019).” Environmental Science & Technology 56, no. 1: 185–193. 10.1021/acs.est.1c02837. 34932322

[eva70317-bib-0034] Hansen, T. F. , C. Pélabon , and D. Houle . 2011. “Heritability Is Not Evolvability.” Evolutionary Biology 38, no. 3: 258–277. 10.1007/s11692-011-9127-6.

[eva70317-bib-0035] Hedrick, P. W. 2001. “Conservation Genetics: Where Are We Now?” Trends in Ecology & Evolution 16, no. 11: 629–636. 10.1016/S0169-5347(01)02282-0.

[eva70317-bib-0036] Hochachka, P. W. , and G. N. Somero . 2002. Biochemical Adaptation: Mechanism and Process in Physiological Evolution. Oxford University Press.

[eva70317-bib-0037] Hofmeister, R. J. , D. M. Ribeiro , S. Rubinacci , and O. Delaneau . 2023. “Accurate Rare Variant Phasing of Whole‐Genome and Whole‐Exome Sequencing Data in the UK Biobank.” Nature Genetics 55, no. July: 1243–1249. 10.1038/s41588-023-01415-w. 37386248 PMC10335929

[eva70317-bib-0038] Holderegger, R. , U. Kamm , and F. Gugerli . 2006. “Adaptive vs. Neutral Genetic Diversity: Implications for Landscape Genetics.” Landscape Ecology 21, no. 6: 797–807. 10.1007/s10980-005-5245-9.

[eva70317-bib-0039] Hudson, N. L. , G. T. Kwan , B. E. Davis , et al. 2026. “Evaluating Stress Recovery and Gill Morphology During Experimental Supplementation of an Endangered Fish Species.” Canadian Journal of Fisheries and Aquatic Sciences 83: 1–19. 10.1139/cjfas-2024-0390.

[eva70317-bib-0041] Hung, T. C. , B. G. Hammock , M. Sandford , et al. 2022. “Temperature and Salinity Preferences of Endangered Delta Smelt ( *Hypomesus transpacificus* , Actinopterygii, Osmeridae).” Scientific Reports 12, no. 1: 1–11. 10.1038/s41598-022-20934-w. 36192440 PMC9530165

[eva70317-bib-0040] Hung, T. , M. Rosales , T. Kurobe , et al. 2019. “A Pilot Study of the Performance of Captive‐Reared Delta Smelt *Hypomesus transpacificus* in a Semi‐Natural Environment.” Journal of Fish Biology 95, no. 6: 1517–1522. 10.1111/jfb.14162. 31613989 PMC6916271

[eva70317-bib-0042] Ignatz, E. H. , M. S. Allen , J. R. Hall , et al. 2025. “Application of Genomic Tools to Study and Potentially Improve the Upper Thermal Tolerance of Farmed Atlantic Salmon ( *Salmo salar* ).” BMC Genomics 26, no. 1: 294. 10.1186/s12864-025-11482-4. 40128646 PMC11934803

[eva70317-bib-0043] Jeffries, K. M. , R. E. Connon , B. E. Davis , et al. 2016. “Effects of High Temperatures on Threatened Estuarine Fishes During Periods of Extreme Drought.” Journal of Experimental Biology 219: 1705–1716. 10.1242/jeb.134528. 27252456

[eva70317-bib-0044] Jenkins, N. L. , C. M. Sgró , and A. A. Hoffmann . 1997. “Environmental Stress and the Expression of Genetic Variation.” In Environmental Stress, Adaptation and Evolution, edited by R. Bijlsma and V. Loeschcke , vol. 83, 79–96. Birkhäuser Basel. 10.1007/978-3-0348-8882-0_5. 9342844

[eva70317-bib-0045] Komoroske, L. M. , R. E. Connon , J. Lindberg , et al. 2014. “Ontogeny Influences Sensitivity to Climate Change Stressors in an Endangered Fish.” Conservation Physiology 2: 1–13. 10.1093/conphys/cou008. PMC4806739 27293629

[eva70317-bib-0046] Kruuk, L. E. 2004. “Estimating Genetic Parameters in Natural Populations Using the ‘Animal Model’.” Philosophical Transactions of the Royal Society of London. Series B, Biological Sciences 359, no. 1446: 873–890. 10.1098/rstb.2003.1437. 15306404 PMC1693385

[eva70317-bib-0047] LaCava, M. E. F. , J. S. Griffiths , L. Ellison , E. W. Carson , T. C. Hung , and A. J. Finger . 2023. “Loss of Plasticity in Maturation Timing After Ten Years of Captive Spawning in a Delta Smelt Conservation Hatchery.” Evolutionary Applications 16, no. 11: 1845–1857. 10.1111/eva.13611. 38029063 PMC10681455

[eva70317-bib-0048] Laikre, L. , M. K. Schwartz , R. S. Waples , and N. Ryman . 2010. “Compromising Genetic Diversity in the Wild: Unmonitored Large‐Scale Release of Plants and Animals.” Trends in Ecology & Evolution 25, no. 9: 520–529. 10.1016/j.tree.2010.06.013. 20688414

[eva70317-bib-0049] Lande, R. , and S. Arnold . 1983. “The Measurement of Selection on Correlated Characters.” Evolution; International Journal of Organic Evolution 37, no. 6: 1210–1226. 10.2307/2408842. 28556011

[eva70317-bib-0050] Lindberg, J. C. , G. Tigan , L. Ellison , T. Rettinghouse , M. M. Nagel , and K. M. Fisch . 2013. “Aquaculture Methods for a Genetically Managed Population of Endangered Delta Dmelt.” North American Journal of Aquaculture 75, no. 2: 186–196. 10.1080/15222055.2012.751942.

[eva70317-bib-0051] Lorenzen, K. , M. C. M. Beveridge , and M. Mangel . 2012. “Cultured Fish: Integrative Biology and Management of Domestication and Interactions With Wild Fish.” Biological Reviews 87, no. 3: 639–660. 10.1111/j.1469-185X.2011.00215.x. 22221879

[eva70317-bib-0052] Lynch, M. , and B. Walsh . 1998. Genetics and the Analysis of Quantitative Traits. *Sunderland Sinauer* .

[eva70317-bib-0053] Mable, B. K. 2019. “Conservation of Adaptive Potential and Functional Diversity: Integrating Old and New Approaches.” Conservation Genetics 20, no. 1: 89–100. 10.1007/s10592-018-1129-9.

[eva70317-bib-0054] Mcguigan, K. , N. Nishimura , M. Currey , D. Hurwit , and W. A. Cresko . 2010. “Cryptic Genetic Variation and Body Size Evolution in Threespine Stickleback.” Evolution 65, no. 4: 1203–1211. 10.1111/j.1558-5646.2010.01195.x. 21463296

[eva70317-bib-0055] McGuigan, K. , and C. M. Sgrò . 2009. “Evolutionary Consequences of Cryptic Genetic Variation.” Trends in Ecology & Evolution 24, no. 6: 305–311. 10.1016/j.tree.2009.02.001. 19328588

[eva70317-bib-0056] McKenna, A. , M. Hanna , E. Banks , et al. 2010. “The Genome Analysis Toolkit: A MapReduce Framework for Analyzing Next‐Generation DNA Sequencing Data.” Genome Research 20, no. 9: 1297–1303. 10.1101/gr.107524.110. 20644199 PMC2928508

[eva70317-bib-0057] Michalak, P. , L. Kang , M. F. Schou , H. R. Garner , and V. Loeschcke . 2019. “Genomic Signatures of Experimental Adaptive Radiation in Drosophila.” Molecular Ecology 28, no. 3: 600–614. 10.1111/mec.14917. 30375065

[eva70317-bib-0058] Moore, M. P. , H. H. Whiteman , and R. A. Martin . 2019. “A Mother's Legacy: The Strength of Maternal Effects in Animal Populations.” Ecology Letters 22: 1620–1628. 10.1111/ele.13351. 31353805

[eva70317-bib-0059] NatureServe . 2014. “*Hypomesus transpacificus* (Errata Version Published in 2020). The IUCN Red List of Threatened Species (Version e.T10722A174778740) [Dataset].” 10.2305/IUCN.UK.2014-3.RLTS.T10722A174778740.en.

[eva70317-bib-0060] Park, J. , and D. Ray . 2026. “A Robust Pleiotropy Method With Applications to Lipid Traits and to Inflammatory Bowel Disease Subtypes With Sample Overlap.” Human Genetics and Genomics Advances 7, no. 1: 100501. 10.1016/j.xhgg.2025.100501. 40913314 PMC12547305

[eva70317-bib-0061] Paul, K. , P. Pélissier , L. Goardon , et al. 2023. “Maternal and Genetic Effects on Embryonic Survival From Fertilization to Swim Up Stage and Reproductive Success in a Farmed Rainbow Trout Line.” Aquaculture Reports 29: 101523. 10.1016/j.aqrep.2023.101523.

[eva70317-bib-0062] Perry, G. M. L. , C. M. Martyniuk , M. M. Ferguson , and R. G. Danzmann . 2005. “Genetic Parameters for Upper Thermal Tolerance and Growth‐Related Traits in Rainbow Trout ( *Oncorhynchus mykiss* ).” Aquaculture 250, no. 1–2: 120–128. 10.1016/j.aquaculture.2005.04.042.

[eva70317-bib-0063] Peterson, R. A. 2021. “Finding Optimal Normalizing Transformations via bestNormalize.” R Journal 13, no. 1: 310–329. 10.32614/rj-2021-041.

[eva70317-bib-0064] Pörtner, H.‐O. , C. Bock , and F. C. Mark . 2017. “Oxygen‐ and Capacity‐Limited Thermal Tolerance: Bridging Ecology and Physiology.” Journal of Experimental Biology 220, no. 15: 2685–2696. 10.1242/jeb.134585. 28768746

[eva70317-bib-0065] Prunier, J. , A. Lemaçon , A. Bastien , et al. 2019. “LD‐Annot: A Bioinformatics Tool to Automatically Provide Candidate SNPs With Annotations for Genetically Linked Genes.” Frontiers in Genetics 10: 1–8. 10.3389/fgene.2019.01192. 31850063 PMC6889475

[eva70317-bib-0066] Rost‐Komiya, B. , D. C. H. Metzger , R. J. Penman , T. S. Blanchard , M. L. Earhart , and P. M. Schulte . 2026. “Thermal Acclimation Affects the Repeatability of Upper Thermal Tolerance in Atlantic Killifish ( *Fundulus heteroclitus* ).” Journal of Thermal Biology 139: 104513. 10.1016/j.jtherbio.2026.104513. 42308783

[eva70317-bib-0067] Rubinacci, S. , D. M. Ribeiro , R. J. Hofmeister , and O. Delaneau . 2021. “Efficient Phasing and Imputation of Low‐Coverage Sequencing Data Using Large Reference Panels.” Nature Genetics 53, no. 1: 120–126. 10.1038/s41588-020-00756-0. 33414550

[eva70317-bib-0068] Satoh, T. 2020. “Diverse Physiological Functions and Regulatory Mechanisms for Signal‐Transducing Small GTPases.” International Journal of Molecular Sciences 21, no. 19: 7291. 10.3390/ijms21197291. 33023216 PMC7583808

[eva70317-bib-0069] Swanson, C. , T. Reid , P. S. Young , and J. J. Cech . 2000. “Comparative Environmental Tolerances of Threatened Delta Smelt ( *Hypomesus transpacificus* ) and Introduced Wakasagi ( *H. nipponensis* ) in an Altered California Estuary.” Oecologia 123, no. 3: 384–390. 10.1007/s004420051025. 28308593

[eva70317-bib-0070] Tobler, R. , S. U. Franssen , R. Kofler , et al. 2014. “Massive Habitat‐Specific Genomic Response in *D. melanogaster* Populations During Experimental Evolution in Hot and Cold Environments.” Molecular Biology and Evolution 31, no. 2: 364–375. 10.1093/molbev/mst205. 24150039 PMC3907058

[eva70317-bib-0071] Tsai, Y. J. J. , L. Ellison , T. Stevenson , W. J. Mulvaney , E. W. Carson , and T. C. Hung . 2022. “Evaluating the Performance of a Small‐Scale Culture System for Delta Smelt.” North American Journal of Aquaculture 84, no. 3: 370–380. 10.1002/naaq.10247.

[eva70317-bib-0072] U.S. Fish and Wildlife Service . 2020. “Delta Smelt Supplementation Strategy (Issue October, pp. 1–55)”.

[eva70317-bib-0073] Utter, F. , and J. Epifanio . 2002. “Marine Aquaculture: Genetic Potentialities and Pitfalls.” Reviews in Fish Biology and Fisheries 12: 59–77.

[eva70317-bib-0074] Via, S. , and R. Lande . 1985. “Genotype‐Environment Interaction and the Evolution of Phenotypic Plasticity.” Evolution 39, no. 3: 505–522. 10.1111/j.1558-5646.1985.tb00391.x. 28561964

[eva70317-bib-0075] Whalen, A. , R. Ros‐Freixedes , D. L. Wilson , G. Gorjanc , and J. M. Hickey . 2018. “Hybrid Peeling for Fast and Accurate Calling, Phasing, and Imputation With Sequence Data of Any Coverage in Pedigrees.” Genetics Selection Evolution 50, no. 1: 1–15. 10.1186/s12711-018-0438-2. PMC6299538 30563452

[eva70317-bib-0076] Wood, C. M. , and D. G. McDonald . 1997. Global Warming: Implications for Freshwater and Marine Fish. Cambridge University Press.

[eva70317-bib-0077] Yanagitsuru, Y. R. , B. E. Davis , and M. R. Baerwald . 2022. “Using Physiology to Recover Imperiled Smelt Species.” In Conservation Physiology for the Anthropocene – A Systems Approach Part B. Elsevier Inc. 10.1016/bs.fp.2022.04.012.

[eva70317-bib-0078] Yeaman, S. 2015. “Local Adaptation by Alleles of Small Effect.” American Naturalist 186, no. S1: S74–S89. 10.1086/682405. 26656219

[eva70317-bib-0079] Zhou, X. , and M. Stephens . 2012. “Genome‐Wide Efficient Mixed‐Model Analysis for Association Studies.” Nature Genetics 44, no. 7: 821–824. 10.1038/ng.2310. 22706312 PMC3386377

