# Supplementary material for: Quantifying the Evolutionary Potential for Delta Smelt Persistence in a Warming Habitat
Source: Evol Appl. 2026 Sep 1;19(9):e70317. doi: 10.1111/eva.70317 (PMC13531369; doi:10.1111/eva.70317)
Supplement: Supplementary file 1 [file EVA-19-e70317-s002.docx]

Figure S1: Factorial breeding block design and offspring rearing temperature experimental design. Families were generated from adult smelt of low, medium, or high DI category. Each block consisted of 5 males (numbers) crossed with 2 females (letters) from the same DI category. Each family was designated by the number and letter combo of its parents (i.e., C10). There were 4 blocks for the low DI group, 3 blocks for the medium DI group, 3 blocks for the high DI group, and 3 blocks which included a “mix” of low, medium, and high DI fish. There were a total of 88 parents crossed and 130 families generated. Each family was evenly split into four groups and reared at either 15C or 18C (with replicates for each temperature). Fish from the same DI category (low, medium, high or mix) were kept together in a single tank, with four tanks fed by a single header tank for each temperature and replicate (i.e., Closed recirculating “system”).

Figure S2: Box plot distribution of each individual offspring’s numerical domestication index (DI) number in each low and high DI category. In FCCL, low DI fish are defined as having a numerical DI below 7, medium between 7 and 10, and high DI fish above 10. Box plot displays median (middle line of box), the first and third quartile of data (box edges), and full range of data (whiskers). Mean numerical DI in the low group was 6.06, medium group was 8.56, and the high group was 10.96

Figure S3: The r2 correlation between imputed genotypes of a down-sampled individuals to 1x coverage (in minor allele frequency bins) and highly-confident genotype calls from the same individual at 30x coverage. Higher correlations suggest that the imputation methods perform more accurately at higher minor allele frequencies in the sampled population reference panel. We filtered our final reference panel to a minor allele frequency cut-off of 2%, resulting in an imputation accuracy of 80-85%.

Figure S4: (A) Percent recovered offspring per family reared at either 15° C (blue) or 18° C (orange). Each family is designated by its numerical domestication index (DI). Low DI <7, medium DI 7-10, high DI >10. (B) Plasticity of survival (Percent recovered offspring per family) at two different rearing temperatures, 15C and 18C.

Figure S5: Percent recovered offspring per family by the mean family CTMax reared at either 15° C(blue) or 18° C (orange).

Figure S6: Manhattan plot of PLACO+ results (-log pvalue) for SNPs associated with both genotype effects on CTMax and temperature-dependent genotype effects on CTMax. No SNPs passed the genome-wide 0.05 significance threshold after Bonferroni correction.

Figure S7: Manhattan plot of PLACO+ results (-log pvalue) for SNPs associated with both genotype effects on CTMax and genotype effects on DI. No SNPs passed the genome-wide 0.05 significance threshold after Bonferroni correction.

Figure S8: Manhattan plot of PLACO+ results (-log pvalue) for SNPs associated with both genotype effects on fork length and temperature-dependent genotype effects on fork length. No SNPs passed the genome-wide 0.05 significance threshold after Bonferroni correction.

Figure S9: Manhattan plot of PLACO+ results (-log pvalue) for SNPs associated with both genotype effects on CTMax and genotype effects on fork length. No SNPs passed the genome-wide 0.05 significance threshold after Bonferroni correction.

Figure S10: Manhattan plot of PLACO+ results (-log pvalue) for SNPs associated with both temperature-dependent genotype effects on CTMax and temperature-dependent genotype effects on fork length. No SNPs passed the genome-wide 0.05 significance threshold after Bonferroni correction.

Figure S11: Manhattan plot of PLACO+ results (-log pvalue) for SNPs associated with both temperature-dependent genotype effects on CTMax and genotype effects on DI. No SNPs passed the genome-wide 0.05 significance threshold after Bonferroni correction.
